# Anatomy and the type concept in biology show that ontologies must be adapted to the diagnostic needs of research

**DOI:** 10.1186/s13326-022-00268-2

**Published:** 2022-06-27

**Authors:** Lars Vogt, István Mikó, Thomas Bartolomaeus

**Affiliations:** 1 TIB Leibniz Information Centre for Science and Technology, Welfengarten 1B, 30167 Hannover, Germany; 2 Don Chandler Entomological Collection, University of New Hampshire, Durham, NH USA; 3 Institut für Evolutionsbiologie und Ökologie, Universität Bonn, An der Immenburg 1, 53121 Bonn, Germany

**Keywords:** FAIR data, Anatomy, Biomedical ontology, Cluster class, Essentialistic class, Fuzzy set, Ontological definition, Recognition criteria, Diagnostic knowledge, Ontological knowledge

## Abstract

**Background:**

In times of exponential data growth in the life sciences, machine-supported approaches are becoming increasingly important and with them the need for FAIR (Findable, Accessible, Interoperable, Reusable) and eScience-compliant data and metadata standards. Ontologies, with their queryable knowledge resources, play an essential role in providing these standards. Unfortunately, biomedical ontologies only provide ontological definitions that answer *What is it?* questions, but no method-dependent empirical recognition criteria that answer *How does it look?* questions. Consequently, biomedical ontologies contain knowledge of the underlying ontological nature of structural kinds, but often lack sufficient diagnostic knowledge to unambiguously determine the reference of a term.

**Results:**

We argue that this is because ontology terms are usually textually defined and conceived as essentialistic classes, while recognition criteria often require perception-based definitions because perception-based contents more efficiently document and communicate spatial and temporal information—a picture is worth a thousand words. Therefore, diagnostic knowledge often must be conceived as cluster classes or fuzzy sets. Using several examples from anatomy, we point out the importance of diagnostic knowledge in anatomical research and discuss the role of cluster classes and fuzzy sets as concepts of grouping needed in anatomy ontologies in addition to essentialistic classes. In this context, we evaluate the role of the biological type concept and discuss its function as a general container concept for groupings not covered by the essentialistic class concept.

**Conclusions:**

We conclude that many recognition criteria can be conceptualized as text-based cluster classes that use terms that are in turn based on perception-based fuzzy set concepts. Finally, we point out that only if biomedical ontologies model also relevant diagnostic knowledge in addition to ontological knowledge, they will fully realize their potential and contribute even more substantially to the establishment of FAIR and eScience-compliant data and metadata standards in the life sciences.

**Supplementary Information:**

The online version contains supplementary material available at 10.1186/s13326-022-00268-2.

## Background

Data exploration has been identified as a new driving force for scientific progress in data-rich fields of empirical research [1]. This new approach to research, also called **eScience↑** (all terms marked **↑** at their first mention are explained in the glossary, listed in their singular form; see supporting information *S**1**Glossary*), developed as a necessary consequence of the emergence of high-throughput technologies and **Big Data↑**. eScience requires the development of applications and services focusing on capture, curation, mining, analysis, and visualization of data and metadata. The applications and services need data and metadata to follow eScience-compliant standards—data must be semantically structured to be **machine-actionable↑** [2–4]. In the context of eScience, data and metadata must be maximally **F**indable, **A**ccessible, **I**nteroperable, and **R**eusable by humans and machines alike and thus comply with the **FAIR↑** Guiding Principles [4] to be efficiently usable.

Anatomy^1^ is currently lacking well established FAIR and eScience-compliant data and metadata standards. While anatomy is primarily a descriptive science [7], it receives explanatory relevance by relating its descriptive data to causal theories, as we can see in functional morphology, developmental morphology, and evolutionary morphology.

Analog to biological **taxonomy↑** and nomenclature, one of anatomy’s central functions is to provide a terminology for inventorying different types of anatomical entities. Anatomical terminology serves as a basic **reference↑** system for all supra-molecular biological entities, thereby providing the descriptive framework for the supra-molecular domain of biology. Anatomy plays a central role in the development of knowledge representations for various disciplines in the life sciences [8–12]. Unfortunately, due to the overwhelming phenotypic diversity, the long history of anatomical research, and various disparate traditions of taxon-centered anatomical communities, anatomy lacks a commonly accepted, standardized, and taxon-independent terminology [7, 13, 14]. This not only hampers the cooperation of morphologists, but also limits the overall retrievability of the majority of biological data.

Anatomy **ontologies↑** used in online data repositories can provide the semantic structure required for representing and documenting phenotype data in an eScience-compliant format following the FAIR Guiding Principles [13, 15–21].

Ontologies are **dictionaries↑** that are used for describing a certain reality. They are formal, machine-actionable representations of the types of entities and relations found in a given domain [22]. They consist of terms for **classes↑** (sometimes also called ‘concepts’) and for relations between classes, both with commonly accepted definitions that are formulated in a highly formalized canonical syntax and standardized format, such as the Web Ontology Language (**OWL↑**) serialized to the Resource Description Framework (**RDF↑**) [23]. Ontology terms carry **semantic conceptual contents↑** (i.e., text-based information), and typically do *not* involve **perceptual non-conceptual contents↑** (i.e., media-based information, e.g., images). They have many applications, including (i) tagging bodies of data to make them available for integration, searches, queries, and analyses, (ii) natural language processing, (iii) automated reasoning, (iv) decision-support systems, and (v) indexing images [22, 24].

In anatomy research, ontologies mostly have been used for annotating natural language phenotype descriptions [16, 25–30], and occasionally for image annotation [31–33]. It has been shown that semantics have the potential to provide a new perspective and promising conceptual framework for solving existing theoretical and methodological problems in anatomy-related research. For instance, studying the evolution of structural complexity [34], closing the methodological gap between homology recognition and homology definition [19], automatically detecting and coding dependent characters in phylogenetics [35], and for a semantic approach to numerical tree inference in phylogenetics [20].

Morphologists describe anatomical entities by transforming their personal perceptions into communicable representations by contrasting iconic (i.e., photos, drawings, diagrams etc.) and textual representations of reality. In this paper, using anatomy as an example, we discuss the concept of **essentialistic class↑** as a way of grouping material entities, as it is commonly used by biomedical ontologies. We discuss the difference between **ontological definition↑** (*What is it?*) and **empirical recognition criteria↑** (*How does it look?*) and point to the importance of unambiguous reference of a term—something that is usually ignored by biomedical ontologies. We argue for the necessity of **cluster classes↑** and **fuzzysets↑** as additional concepts of grouping in biomedical ontologies, which are required to cover diagnostic knowledge in addition to the ontological knowledge already covered by essentialistic classes. We discuss how the biological type concept relates to these three concepts of grouping and, finally, address the tension between semantic conceptual contents and perceptual contents and evaluate the applicability of biomedical ontologies in everyday anatomical research practice.

## Methods

### Reality and its representations

#### Distinguishing real entities from their cognitive, iconic, and textual representations

In science, we are dealing with **real entities↑**. Real entities (i.e., objects, processes, qualities, and states) are entities that exist in reality, independent of any human mind [36, 37]. Any given real entity is either a **universal↑** (**kind↑**, type) or a **particular↑** (individual, token) [38, 39]. Whereas a universal is multiply located, a particular is always bound to a specific location in space and time. One can define a universal as anything that is instantiated by some particular and a particular as anything that instantiates some universal [40]. Examples of universals include *human being*, the chemical element *Pb*, and *cell*. Examples of particulars include *you* and *me*, *this specific instance of Pb*, and *one of your cells*. A particular anatomical entity is a real entity that is either an individual organism or a part of an individual organism (cf. ‘anatomical entity’ of *Uber Anatomy Ontology*; http://purl.obolibrary.org/obo/UBERON_0001062).

We refer to real entities using three different types of representations.

1. **Cognitive representations↑** are thoughts, perceptions, conceptions, ideas, and beliefs about a given real entity (universal or particular) in the mind of a person. Everyone uses cognitive representations to create a mental model of their environment.

We use two types of **representational artifacts↑** when communicating with others about real entities and our cognitive representations of them. The purpose of representational artifacts is to induce cognitive representations in the receiver that resemble those held by the sender—we want to share the same cognitive representation when communicating about the same real entity [36, 37].

2) **Iconic representational artifacts↑** are, e.g., images, image stacks, 3D models, video, and audio recordings of real entities. Such media items carry perceptual non-conceptual contents (i.e., image-based or audio-based information) that do not denote but rather demonstrate (see *aesthetic non-conceptual content* [36]). The meaning contained in a media item is not arbitrary but rests on a natural relation of resemblance to the part of reality that it reproduces (*natural meaning* [41, 42]).

Media items carry semantically unprocessed information and are not open to direct discourse. If no additional information is associated with them, they become increasingly inaccessible as the number of media items stored in a repository increases, ultimately turning the repository into a media cemetery. Media items are important for documenting scientific results because they carry complex spatial and temporal information that often cannot be represented using **textual representational artifacts↑** (but see [43]). Moreover, contrary to words, a media item can—based on its natural relation of resemblance—carry meaning independent of other representational artifacts, and thus can possess validity by itself [44]. Media items can also increase the trustworthiness of data by functioning as objective proofs [45, 46]. Iconic representational artifacts thus take in a *media*ting role between real entities and their textual representational artifacts. However, they contain information that must be transferred into a textual representation to become data [36].

3) Textual representational artifacts represent cognitive representations that have been translated into a publicly accessible and enduring form, for instance, in sentences that contain **propernames↑**, alphanumeric identifiers, and **kind terms↑**. Textual representational artifacts carry semantic conceptual content (i.e., text-based information) by using words. Words, in turn, are linguistic conventions and their meaning is determined by common agreement [36] (*non-natural meaning* [41]). We can distinguish between textual representational artifacts of particulars in the form of proper names such as the *Large Hadron Collider* or *Bob* and of universals in the form of kind terms such as *particle accelerator* or *Homo sapiens* [36, 47].

Categories such as *true* and *false*, as well as basic modes of reasoning such as deduction and induction, can be applied only to semantic conceptual content. Textual representational artifacts are directly open to discourse. Consequently, data must take the form of textual representational artifacts.

Textual representational artifacts can be represented as **semantic graphs↑**. A semantic graph is a network of RDF/OWL-based **triple↑** statements. RDF is a knowledge representation language developed for representing relationships between (Web) resources in the form of Uniform Resource Identifiers (**URI↑**s; for reasons of human readability, we use labels instead of URIs in this paper to refer to resources). An RDF triple statement consists of *Subject*-*predicate*-*Object*. *Subject* represents the object to be described, *predicate* one of its properties to be described or a relation in which it stands to another object, and *Object* either the value of the property or another object to which it has the relation specified by the *predicate*. In a semantic graph, a URI can take the *Object* position in one triple and the *Subject* position in another triple, connecting the triples to form a directed labeled graph.

#### Four types of textual representational artifacts

We must distinguish four different types of textual representational artifacts and their corresponding semantic conceptual contents [22, 48, 49]:

1. **Universal statements↑** are statements that are true for all **instances↑** of a specific universal. In **Description Logics↑**, they are referred to as TBox expressions and can be documented as class-based semantic graphs using RDF/OWL. Universal statements represent commonly accepted domain knowledge. Definitions of anatomical terms, as they are provided in glossaries or in anatomy ontologies in the form of class axioms, are examples for universal statements [49].
2. **Contingent statements↑** are statements that are true for some instances of a specific universal but not necessarily for every instance. Contingent statements express what is typically found in instances of a given class but not necessarily in every instance of that class.
3. **Terminological statements↑** are statements about linguistic items such as synonymy statements in glossaries or ontologies.
4. **Assertional statements↑** are statements about particulars. In Description Logics, they are referred to as ABox expressions and can be documented as instance-based semantic graphs using RDF/OWL. A **‘factual’ description↑** is a specific type of assertional statement that summarizes the results of an observation about a particular. Everybody who reads a ‘factual’ description should receive the same or very similar cognitive representations of the described real entity [3, 36]. Only ‘factual’ descriptions qualify as data, as they represent observations recorded using words, numbers, and symbols. Terms used in ‘factual’ descriptions can be defined in ontologies as universal and terminological statements.

Assertional and contingent statements should be organized as **semantic knowledge graphs↑** and stored separately from ontologies [49]. Universal statements provided by anatomy ontologies function as referents of data in a semantic knowledge graph, and ontologies are used to meaningfully structure and organize ‘factual’ descriptions in a semantic knowledge graph. Without the connection between data in semantic knowledge graphs in the form of assertional and contingent statements and the ontology terms they reference, data would be neither retrievable nor comprehensible and comparable [19, 20], whereas the combination provides the required semantic structure for documenting FAIR data and metadata [21].

#### Not just names: ordering and classifying biomedical diversity

**Fig. 1:**
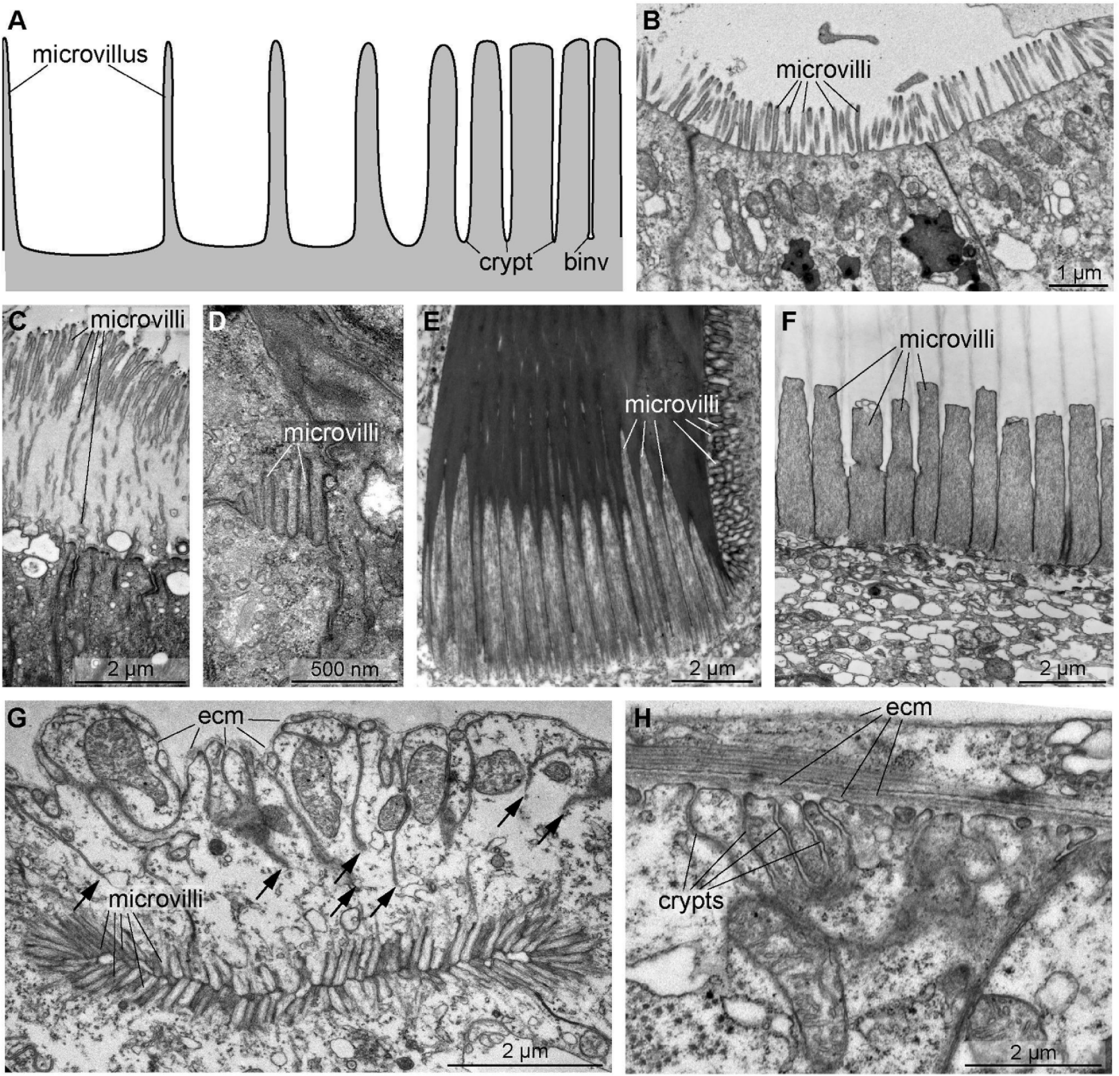
Continuity between microvilli (evaginations) and crypts (invaginations) and examples from different tissues. **A** Scheme of the continuum between microvilli and crypts (modified from [50]). **B** Microvilli of the gut epithelium in *Artemia salina* (Crustacea), metanauplius. **C** Epidermal microvilli in *Owenia fusiformis* (Annelida). **D** Microvilli in a developing rhabdomeric photoreceptor cell in *Achelia brevicauda* (Pantopoda). **E** Microvilli of a chaetoblast during chaetogenesis in *Johnstonia clymenoides* (Annelida). **F** Stereovilli of a chaetoblast during chaetogenesis in *Echiurus echiurus* (Annelida). **G** Duct of the antennal nephridium in *Artemia salina* (Crustacea). *Arrows* mark basal invaginations of the cell membrane. **H** Basal crypts of gut cells in *Artemia salina* (Crustacea)

**Fig. 2:**
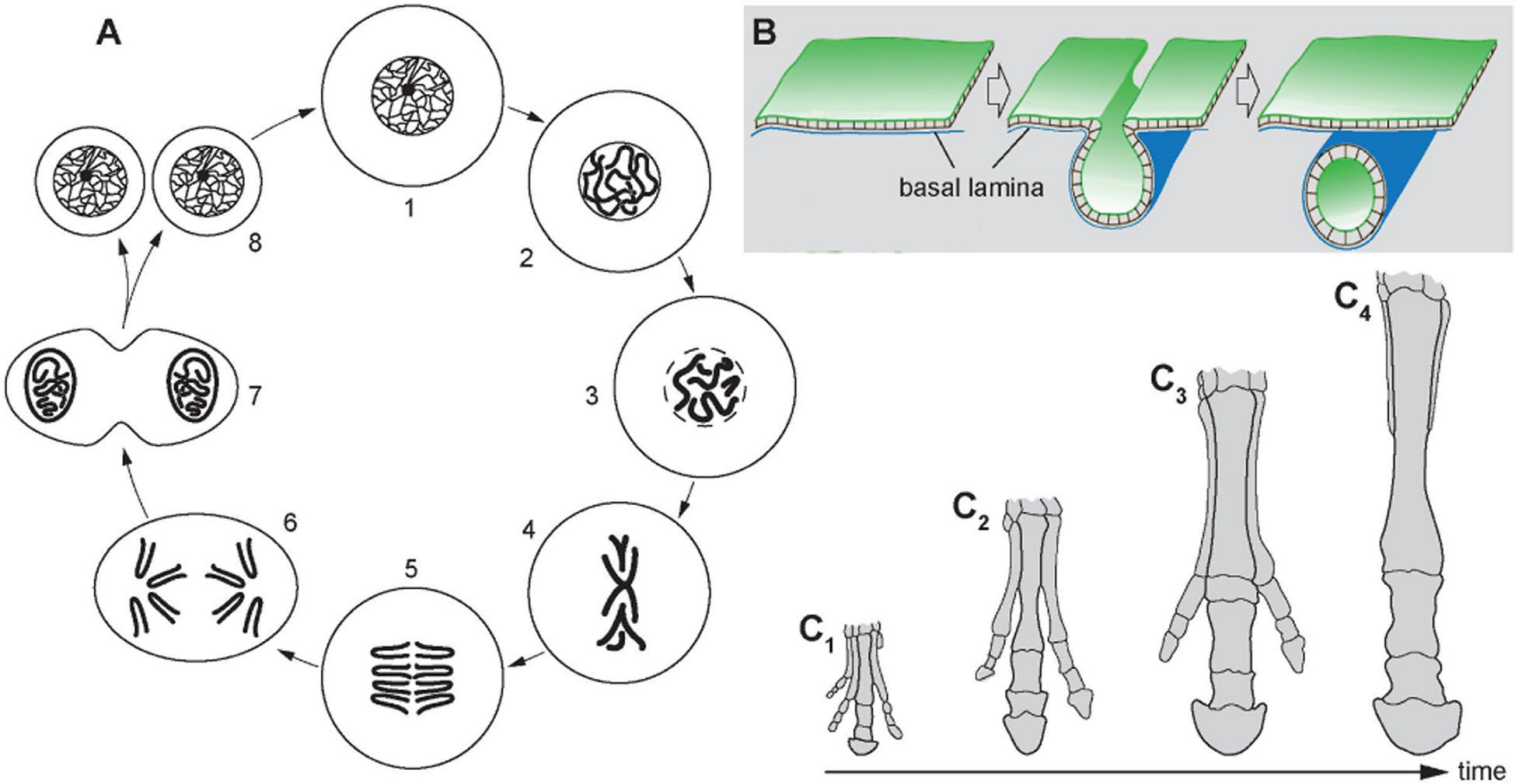
Continuity of forms in time. **A** Cytology. Cell cycle, after [51], modified by excluding centrioles and microtubules. Supercoiling of the DNA leads to chromosome condensation during the prophase and re-condensation during late anaphase and telophase. Since a nuclear membrane is considered to be constitutional for the cell nucleus and this membrane is absent from the metaphase to the early telophase, a cell nucleus is not present during these phases. *1* end of S-phase, *2* prophase, *3* pro-metaphase, *4* metaphase, *5* anaphase, *6* late anaphase, *7* telophase, *8* start of S-phase. **B** Embryology. Neurulation in the chordate development. Ectodermal cells form a fold by differential contraction of the apical actin filament belt. This fold becomes tube-like, detaches from the ectoderm, and forms an epithelial tube underneath the ectoderm. The tube develops into the medullary cord of the central nervous system. *Blue* basal lamina, *green* epithelial surface. **C** Evolution. Formation of the fore-limb of the horse (redrawn from different sources). During the course of evolution, the third toe becomes re-enforced, while toes 1, 2, 4, and 5 are successively reduced (C1 †*Hyracotherium leporinum*, C2 †*Mesohippus biardi*, C3 †*Merychippus insignis*, C4 *Equus ferus*)

For being able to generalize over the structural variety of biomedical entities, we must order and classify them. Ordering biological entities into **linear sequences↑** can be based on their spatio-structural properties such as a continuum of forms between two extremes (Fig. 1) or on their spatiotemporal properties such as the continuum of interactions (Fig. 2A), the continuum of forms during developmental processes (Fig. 2B), or the continuum of forms during evolutionary transformations (Fig. 2C). In general, objects and processes can also be ordered according to numerical properties such as alphabetical sequences, chronological sequences, flow-charts, or bestseller lists [52].

Ordering biological entities into **general groups↑** (sets or classes) represents the most commonly used way to efficiently manage representational artifacts. A **set↑** is defined extensionally by listing its elements (i.e., denotative definition), whereas a class is always defined intensionally by a predicate (i.e., connotative definition) [53, 54]. Class definitions are universal or contingent statements that carry semantic conceptual content. Instances of a class possess the predicate that defines the class. The instances of a class, thus, do not have to resemble each other in all their properties.

Classes as such are concepts and not real entities. If a class possesses a real correlate, one can use the term ‘class’ for referring to the concept, the term ‘kind’ for referring to the real entities, and the term ‘kind term’ for referring to the word we use to refer to the class and the kind. Following this notion, kinds of biological objects or processes exist, while classes are our cognitive representations of them and kind terms our textual representational artifacts for them.

### Aristotelian definitions and Essentialistic classes

When we use a kind term, we use it with an associated intended meaning in mind—its **semantic value↑**—which should resemble the cognitive representation of the sender [55]. A semantic value can be specified by a class definition. The class definitions of biomedical ontologies are usually **Aristotelian definitions↑** [39]. Aristotelian definitions represent universal statements and always consist of two parts, **genus↑** and **differentia↑** [13, 14]. Genus specifies the essential properties of the **parent class↑** that any of its instances must necessarily possess. These properties are inherited downstream from the parent class to all its **subclasses↑**. Differentia specify the essential properties of the class itself and is required for distinguishing it against all its **sister-classes↑**. Only the combination of genus and differentia is sufficient for unambiguously determining membership to a defined class.

Classes that are defined using Aristotelian definitions are called essentialistic classes, and they are based on universal statements. Membership to an essentialistic class is determined in dependence on a set of properties that are individually necessary and jointly sufficient [56]. Therefore, membership of a given particular to an essentialistic class is either *yes* or *no*. Classifying kinds into essentialistic classes results in a hierarchical system of kind terms that can be represented as a tree and forms a taxonomy [57]. Due to their clarity and simplicity, essentialistic classes are well suited for ontologies.

**Fig. 3:**
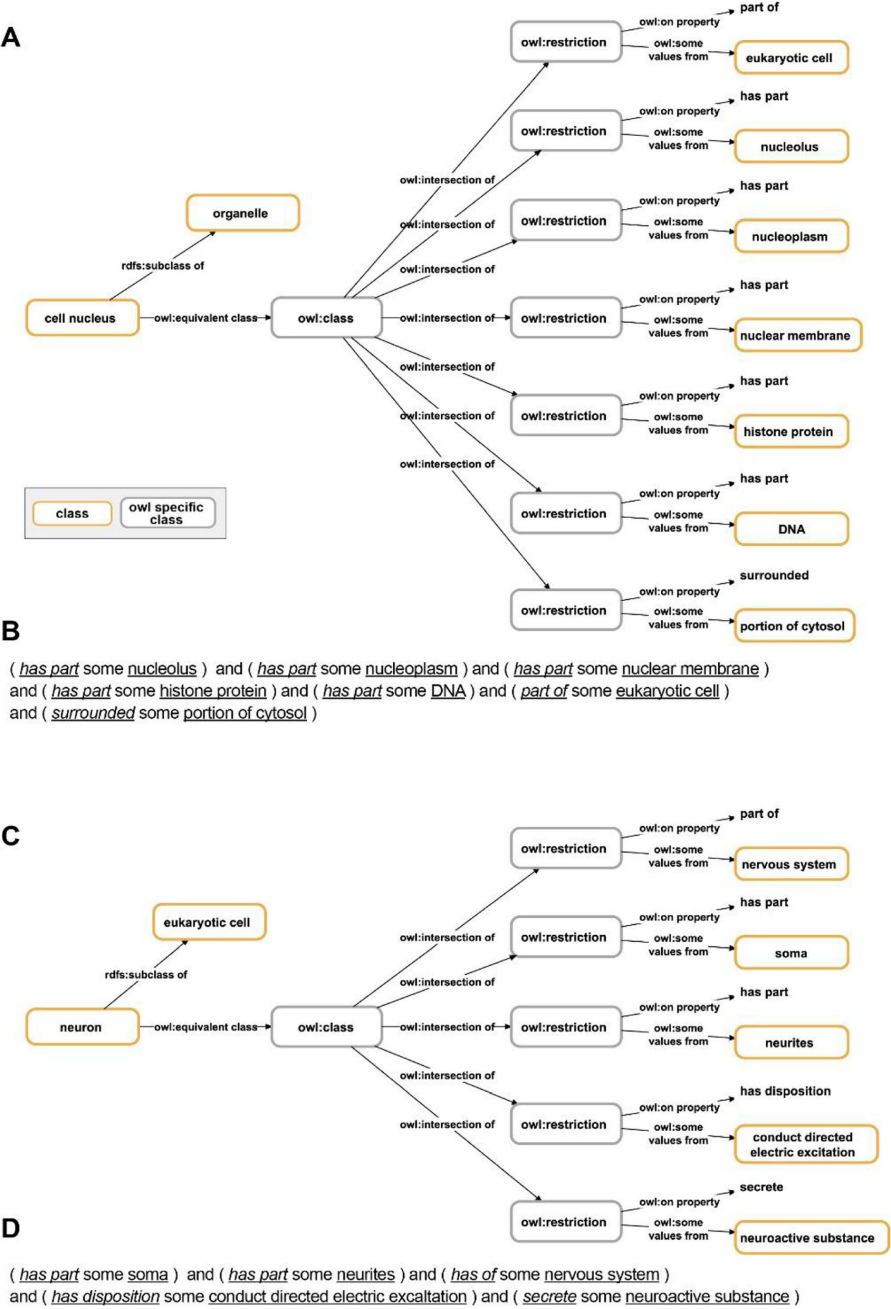
Logical and machine-actionable formal definitions using OWL graph syntax and **OWL Manchester syntax↑** (compare with the human-readable definitions in Table 1). **A** The logical machine-actionable formal definition of the class ‘cell nucleus’ using OWL graph syntax. **B** The same definition in OWL Manchester syntax which is easier to read for a human reader. **C** The logical machine-actionable formal definition of the class ‘neuron’ using OWL syntax. **D** The same definition in OWL Manchester syntax. All definitions based on [58]

Ontologies should provide Aristotelian definitions in two different formats, as human-readable natural language definitions (Table 1) and as logical and machine-actionable formal definitions in the form of class axioms (Fig. 3) [22].

**Table 1:** Natural language Aristotelian definitions of four different types of anatomical entities

*cell nucleus*	*“Organelle which has as its direct parts a nuclear membrane and nuclear matrix”* (Foundational Model of Anatomy, http://purl.org/sig/ont/fma/fma63840)
*nucleated cell*	*“Cell which has as its direct part a maximally connected part of protoplasm.”* (Foundational Model of Anatomy, http://purl.org/sig/ont/fma/fma67513)
*neuron*	*“A neuron is a* [eukaryotic] *cell. It is part of the nervous system and consists of a soma that gives rise to neurites, which conduct electric excitation in a directed way. A neuron communicates with other cells* via *synapses. Most neurons synthesize and secret neuroactive substances.”* ([58], p.23)
*receptor cell*	*“A receptor cell is a neuron. It is part of the nervous system. In a signal transduction chain, it is the first neuron that converts an adequate stimulus into an electric signal.”* ([58], p.34)
*globuli cell*	*“A globuli cell is a neuron. It is part of a cluster of other globuli cells. It possesses a minute amount of cytoplasm and a nucleus containing condensed chromatin. The somata of globuli cells are densely packed and easily discernible* … *due to their small diameter”* ([58], p.14)

**Fig. 4:**
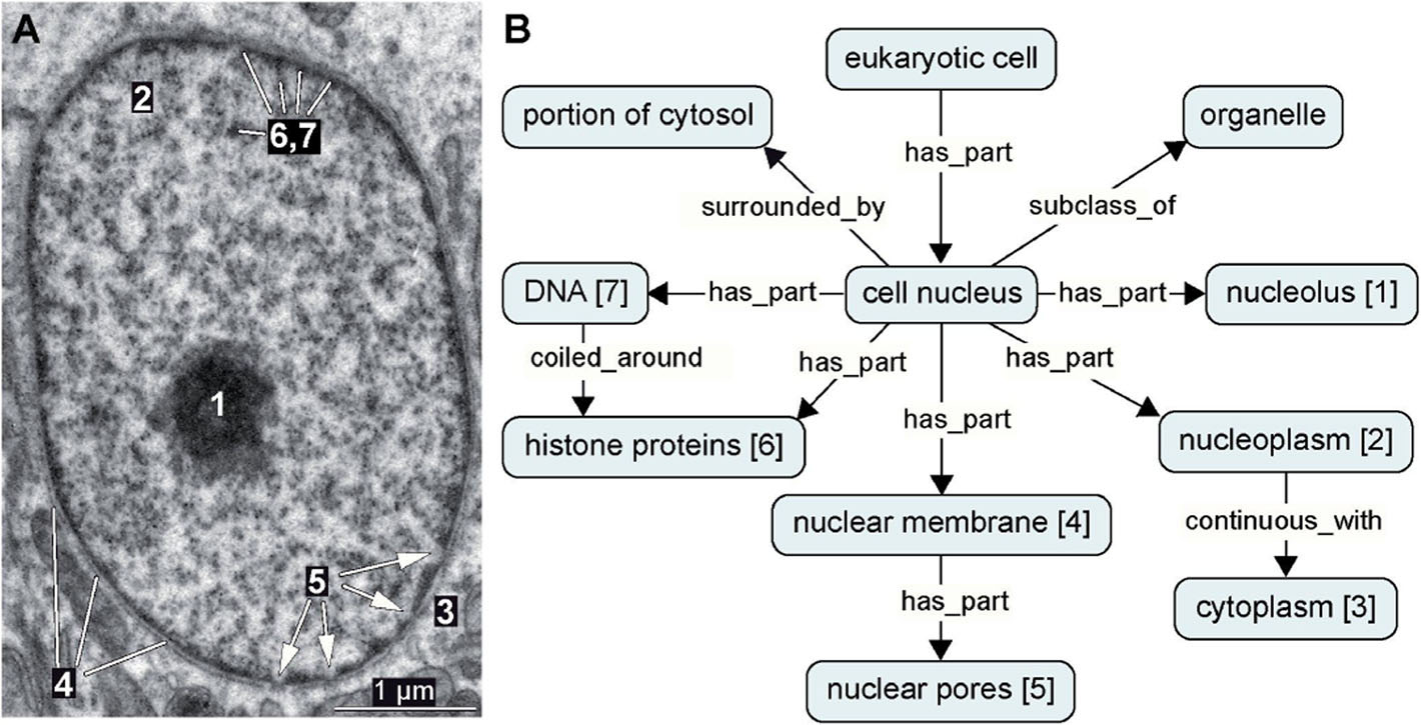
*Lumbrineris tetraura* (Annelida), nucleus of follicle cell. **A** Transmission electron microscopy, transverse section. Numbering corresponds to **B***1* nucleolus, *2* nucleoplasm, *3* cytoplasm, *4* nuclear membrane, *5* nucleoplasm, *6* histone proteins, *7* DNA. Scattered electron-dense material within the nucleus is usually termed chromatin (see 5.2). **B** Graphical representation of RDF statements about the nucleus and its substructures. References to sub-structures marked in **A** are in brackets

Formal logical definitions require a highly formalized syntax such as provided by RDF. RDF triple statements can be modeled as graphs. When using OWL, which represents a commonly used knowledge representation language that can be serialized to RDF, and when including Description Logics (OWL-DL), one can use RDF’s triple formalism for expressing universal statements and thus machine-actionable Aristotelian definitions (Fig. 3). The same formalism can be used for expressing assertional statements and thus anatomical ‘factual’ descriptions (Fig. 4B). A set of interconnected triples can result in a semantic network, jointly forming a directed labeled graph (Fig. 3, 4B) to which graph logic can be applied [2, 19, 59, 60].

## Results

### Aristotelian definitions versus empirical recognition criteria

An Aristotelian definition offers transparency regarding the semantic value (i.e., meaning) of its associated kind term through an ontological definition and provides an answer to the *What is it?* question. Whereas transparency regarding the meaning of a term is of invaluable importance for scientific communication, being able to unambiguously reference a kind term (i.e., correctly applying a term to a given real entity) is just as important. Unfortunately, Aristotelian definitions often do not provide sufficient information about the epistemological^2^ appearance (**diagnosis↑**) of instances and lack specifications of the diagnostic properties that are required for scientists to successfully recognize and identify particular instances of the corresponding essentialistic class. The appearance, however, depends on the applied methods and techniques. Any knowledge about the ontological nature of a real entity is necessarily epistemological, as it always depends on empirical evidence and a chain of reasoning. Communication requires the specification of empirical recognition criteria that determine the cognitive representation of an entity’s appearance and thus provide answers to *How does it look?* questions.

The distinction between semantic conceptual content and reference and thus between ontological definition and empirical recognition criteria is important not only in biology, but in science in general. These two aspects are not identical, as the example of the *Morning Star* and the *Evening Star* demonstrates (or *Clark Kent* and *Superman*), which are co-referring expressions that refer to the same referent, which is the planet Venus (or a superhero character in a comic), but differ considerably in their semantic conceptual contents [61, 62].

Scientists who must identify particular structures, species, or diseases deal with this situation in their daily routines. For instance, lead (*Pb*) is defined as the element with the atomic number 82. This definition, however, does not provide a scientist with practically applicable criteria for empirically testing whether a given object is lead. A variety of methods and procedures has been developed to test for lead—empirical diagnostic knowledge that often has not been directly derived from the ontological definition. Therefore, for many real entities, our cognitive representations of them must be two-sided, because the ontological nature of a given real entity often does not coincide with its outward appearance.^3^ The cognitive representation of a real entity should cover both its ontological nature and its empirical appearance.

This duality of cognitive representations must be reflected in two corresponding representational artifacts for their communication: 1) a textual ontological definition and 2) method- and instrument-dependent textual or perception-based recognition criteria. Both serve to order things into groups, the underlying idea being that the extension of the epistemological group coincides with that of the ontological group. And therefore, both must apply an appropriate grouping concept such as the essentialistic class concept.

The duality of cognitive representations directly relates to the distinction of inferential and referential lexical competence [64]. **Inferential lexical competence↑** of a person depends on the person’s knowledge about the meaning of a term. Inferential lexical competence can be further differentiated into semantic inferential competence, which relates to natural language and formal logical Aristotelian definitions, and output inferential competence, which relates to the words and phrases (i.e., labels) used for referring to a specific concept [22]. **Referential lexical competence↑** of a person, depends on the person’s knowledge about the typical appearance of instances of a kind, allowing them to recognize a portion of reality based on a given ‘factual’ description. Referential lexical competence can be further differentiated into naming referential competence, which refers to a person’s ability to select the right label (object is given → word must be found), and application referential competence, which refers to a person’s ability to select the right particular object for a given label (word is given → object must be identified) [64]. Whereas inferential lexical competence relies on a semantic system, referential lexical competence relies on a perceptual and motor system. Although these two systems are distinct, they both interact [22].

In the following, we demonstrate using anatomical examples that empirical recognition criteria cannot always be derived from ontological definitions and therefore require a separate specification.

#### Textual recognition criteria

##### Recognizing a cell nucleus

According to its ontological definition (Table 1, Fig. 3), a cell nucleus is a cell organelle with a nucleolus, DNA, histones, and other proteins and a surrounding membrane, and it is located inside the cytoplasm of a eukaryotic cell. Identifying a cell nucleus inside a cell requires more information.

**Fig. 5:**
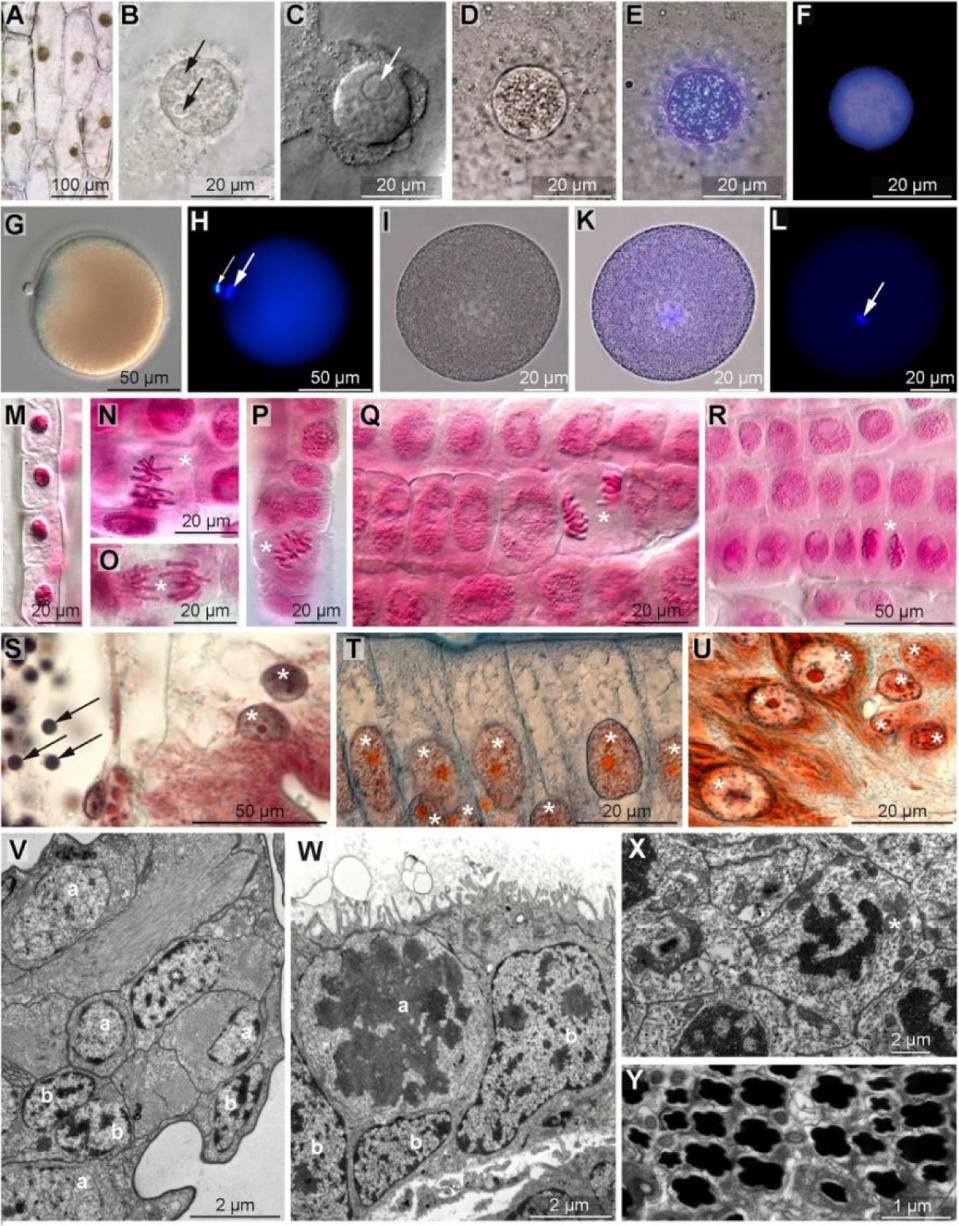
**A**-**F***Allium cepa* (Amaryllidacea, Magnoliopsida), nucleus of epidermis cell visualized using bright-field (**A**, **B**), Nomarski (**C**), phase contrast microscopy (**D**), phase contrast plus fluorescence microscopy (**E**), and fluorescence microscopy (**F**). Nucleus was stained *blue* with Hoechst 33342 in **E** and **F**. Note prominent nucleoli in **B** and **C**. **G**-**L***Riseriellus occultus* (Lineidae, Nemertea), fertilized egg cells. Due to the yolk content, the nucleus cannot be seen, neither with Nomarski contrast (**G**) nor with phase contrast microscopy, without being specifically stained (**I**). Hoechst 33342 stained chromosomes (*blue*, *arrows*) can be seen under a fluorescence microscope (**H**, **L**) that was combined with phase contrast microscopy in **K**. Note the fully condensed chromatin in the polar body (*small arrow* in **H**). **M**-**R***Allium cepa*, root meristem, Nomarski microscopy, nuclei stained in red with Schneider carmine dye (carmine acetic acid). **M** differentiated nucleus. **N**-**R** mitotic stages (*asterisks*), a nuclear membrane is absent. **N** metaphase, **O** anaphase, **P** metaphase, **Q** late anaphase, **R** telophase. **S**-**U** Histological sections, Azan staining. **S***Clymenura clypeata* (Maldanidae, Annelida). Coelom with sperm cells (*arrows*) and adjacent gut epithelium (*asterisks* mark nuclei). **T***Salamander salamander* (Amphibia, Craniota). Gut epithelium (*asterisks* mark nuclei, note red nucleolus). **U***Bufo bufo* (Amphibia, Craniota), tadpole. Medullary cord, ganglion cells (*asterisks* mark nuclei, note *red* nucleolus). **V**-**Y** Transmission electron microscopy, ultrathin (70 nm) sections, uranyl acetate plus lead citrate staining. **V***Achelia brevicauda* (Pantopoda, Arthropoda), Embryo. Euchromatic (*a*) and heterochromatic (*b*) nuclei (*asterisk* marks nucleolus). **W***Epiperipatus biolleyi* (Onychophora, Euarthropoda), embryo, epidermis with mitotic (*a*) and large heterochromatic (*b*) nuclei. **X***Tetrastemma cerasinum* (Nemertea). Meiotic cell (*asterisk*). **Y***Amphiporus lactifloreus* (Nemertea). Sperm nuclei (*black*)

Without any staining techniques, if the tissue examined is unicellular or consists of only a few cell layers, bright-field, Nomarski interference, autofluorescent-based confocal laser scanning microscopy (CLSM), or phase contrast microscopy allows identifying the nucleus as a restricted compartment with a granular inner and a spherical outer shape (Fig. 5A-D; Table 2). The nucleolus can be identified as a more homogenous material with a circular outline. All four light microscopy methods are the most non-invasive methods that can be applied. In phase contrast microscopy, the nucleus appears as a restricted compartment with coarse inner material surrounded by a refracting sheath (Fig. 5D; Table 2), which makes this technique ideal for manipulating the DNA or for in-vitro fertilization.

**Table 2:** Diagnostic criteria of ‘cell nucleus’

***Method***	Recognition Criteria
bright-field, Nomarski contrast microscopy	*nucleus appears as a restricted compartment with a granular inside*
phase contrast microscopy	*nucleus appears as a restricted compartment with coarse inner material surrounded by a refracting sheath*
bright-field microscopy of fixed tissue stained using carmine or hematoxylin	*nucleus appears as a restricted compartment with clustered or completely red (carmine) or black (hematoxylin) pigmentation*
transmission electron microscopy, stained with heavy metals	*nucleus appears as a membrane-bound compartment scattered irregularly with electron-dense clusters of varying size to complete electron-dense staining*

Specific stains that interact with the DNA or with histones are used in fluorescent microscopy (Hoechst, Sytox) and histology (chromatin-specific stains such as carmine, hematoxilin, and others) for identifying the nucleus. Fluorescent stains that are specific for DNA and/or RNA exclusively result in a circular or ovoid coarse structure that emits light if illuminated with the wavelength specific for the chromophore used (Fig. 5; Table 2), irrespective of whether the DNA is unfolded (Fig. 5E, F) or condensed (Fig. 5H, L). Fluorescent microscopy may be combined with other non-invasive methods to localize the nucleus (Fig. 5E), especially when cell transparency is not given, as in yolky egg cells (Fig. 5K). Fixed tissue, if stained with dyes interacting with the histone proteins (Fig. 5M-U), allows identifying the nucleus as restricted compartment clustered or completely stained according to the dye used (Table 2). The nucleolus always shows homogenous staining which often is brighter than the histones.

The affinity of histones to acid staining is used in electron microscopy, where uranyl acetate is applied to visualize chromatin (Fig. 5V-Y). Using electron microscopy, the nucleus can be recognized as a compartment bound by a double layer of biomembranes that regularly line pores so that nucleoplasm and cytoplasm form a continuum (Fig. 4A). This can only be seen on the ultrastructural level. Here, the nucleus is also recognized by its chromatin that is either dispersed (euchromatin) or tightly (heterochromatin) or completely (sperm) packed, which causes irregular coarse, clustered, or complete electron-density after heavy metal staining (Fig. 5V, Y; Table 2). During mitoses, the affinity of the chromatin-specific staining allows identification of nuclear material although the nuclear membrane is absent (Fig. 5W, X). The condensed structure of metaphase chromosomes also allows their identification, even without staining, by using phase contrast or Nomarski microscopy (not shown).

##### Recognizing a neuron

According to its ontological definition, a neuron is defined as consisting of a soma and neurites and as having the disposition to conduct electric excitation across its outer membrane via voltage-dependent ion channels (Table 1, Fig. 3). Identifying an instance of ‘neuron’ therefore requires running experiments, which cannot be run using fixed tissue.

Alternatively, when isolated, neurons can be recognized in cell cultures by their shape using phase contrast or Nomarski microscopy, but histological techniques and electron microscopy are needed when they are clustered in a piece of tissue (Table 3). The soma of a neuron can be identified by its large size, but visualizing neurites differs tremendously between the methods used. Single neurites can be seen when using silver impregnation method (Golgi’s methods or Golgi stain). Perikarya and neurites are dark in these stainings.

**Table 3:** Diagnostic criteria of ‘neuron’

***Method***	Recognition Criteria
phase contrast, Nomarski microscopy	*neuron is identifiable by its composition consisting of a soma and one or more peripheral neurites*
use of fluorochrome-labeled antibodies in fluorescence microscopy	*neuron is either identifiable by its composition or by its components (*i.e.*, specific neurotransmitters)*
transmission electron microscopy	*neurites will be small in diameter and contain small mitochondria, vesicles, microtubules and/or neurofilaments, while the soma of a neuron can only be identified by differential diagnostics*

**Fig. 6:**
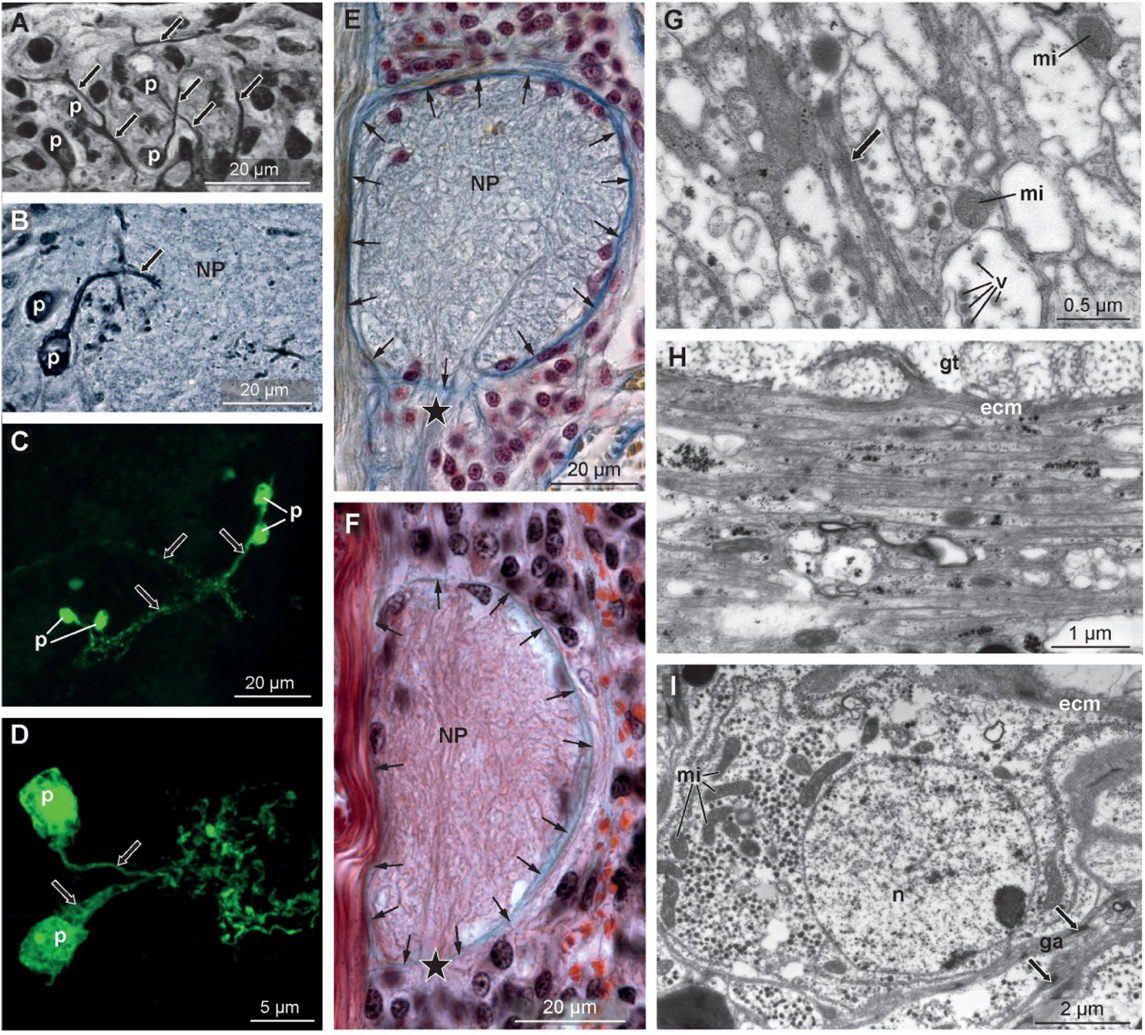
Neurons and nervous system under various stainings. **A**, **B***Lineus viridis* (Nemertea), medullary cord. Silver impregnation. Note that not all neurons of a neuropile (*NP*) are stained (**B**). **C**, **D***Capitella teleta* (Annelida). Anti 5HT with fluorescent secondary antibody. Note that perikaryon and neurite are stained (*green*). Terminal section of neurites is branched (**D**). **E**, **F***Tetrastemma melanocephala* (Nemertea), medullary cord. Azan staining (**E**) and Masson-Goldner-trichrome staining (**F**). Single neurons cannot be discriminated; perikarya of the neurons group mostly dorsal and ventral of the neuropile (*NP*). Neuropile and perikarya are separated by an *ecm* (extracellular matrix) called inner neurilemma (small arrows); groups of neurites can be seen piercing the inner neurilemma (*asterisk*). **G**-**I***Carinina ochracea* (Nemertea), TEM. **G** Transverse section of several neurites. Note small spherical mitochondria (*mi*), neurofilaments (*arrow*), and dense core vesicles (*v*), probably containing serotonin (Trueta et al. 2012). **H** Sagittal section of the cephalic nerve with several neurites. Electron-density of neurites is slightly higher than in surrounding tissue. **I** Perikaryon with large euchromatic nucleus, peripheral neurosecretory vesicles, and large mitochondria. Perikarya are often surrounded by glial cells that are interconnected by desmosomes (*arrow*). *gt* glandular tissue, *p* perikaryon

The perikaryon is a large globular or pear-shaped structure; the nucleus can generally not be discriminated within the perikaryal mass (Fig. 6A). Stronger portions of the neurites are stained, whereas finer branches are often not (Fig. 6B). A specific staining using fluorescent-labeled antibodies against neurotransmitters (serotonin, FMRF-amines, synapsin) or subcellular components (neurotubulin, neurofilaments) visualizes the entire neuron or specific parts within a given piece of tissue (Fig. 6C, D). This technique, however, allows identifying only neurons that contain known neurotransmitters. In AZAN or Masson-Goldner trichome stained histological sections, neuronal tracts can be identified within a brain and neurites be visualized, albeit they cannot be resolved in detail and are weakly stained (Fig. 6). Identification of bundles of neurites is easier if they are surrounded by matrix (small arrows in Fig. 6E, F). Electron microscopy allows resolving neurites in detail. Neurites can be identified by their small diameter, their dense packing, and especially by their subcellular components such as vesicles, neurofilaments, and tiny mitochondria (Fig. 6G, H). Perikarya of neurons contain a large spherical euchromatic nucleus, large mitochondria, and often numerous vesicles in the surrounding cytoplasm (Fig. 6I). In invertebrates, the nucleus may be surrounded by glial cells. In general, however, there is no unambiguous structural component that allows identifying a neuronal perikaryon in the electron microscope without 3-D reconstruction.

##### Recognizing a receptor cell

A receptor cell converts a specific kind of stimulus or sensory input (external stimuli changing the conformation of membrane-bound proteins) into an active physiological event capable of initiating neuronal activity in another cell(s), usually as a change in electric potential. Receptor cells always pass neuronal activity to an effector (i.e., nerve cell, muscle cell, gland cell). The terminology is not uniform and either classifies receptors according to their structure or their function. In general, a receptor cell possesses a specific site to perceive the stimulus, called transducer herein, and a site to transmit the signal to a nerve cell. Concerning their location and anatomy, receptor cells fall into neuronal and epithelial receptor cells.

Neuronal receptor cells or receptor neurons are located in deeper tissue layers. Silver impregnation methods and 3D reconstruction of series of histological or ultrathin sections allow detecting receptor cells light- or electron-microscopically by having a free-end that lacks any connection to a synapse. In vertebrates, they can be recognized by their position (outside the central nervous system) and the absence of any myelin sheath and synapses.

**Fig. 7:**
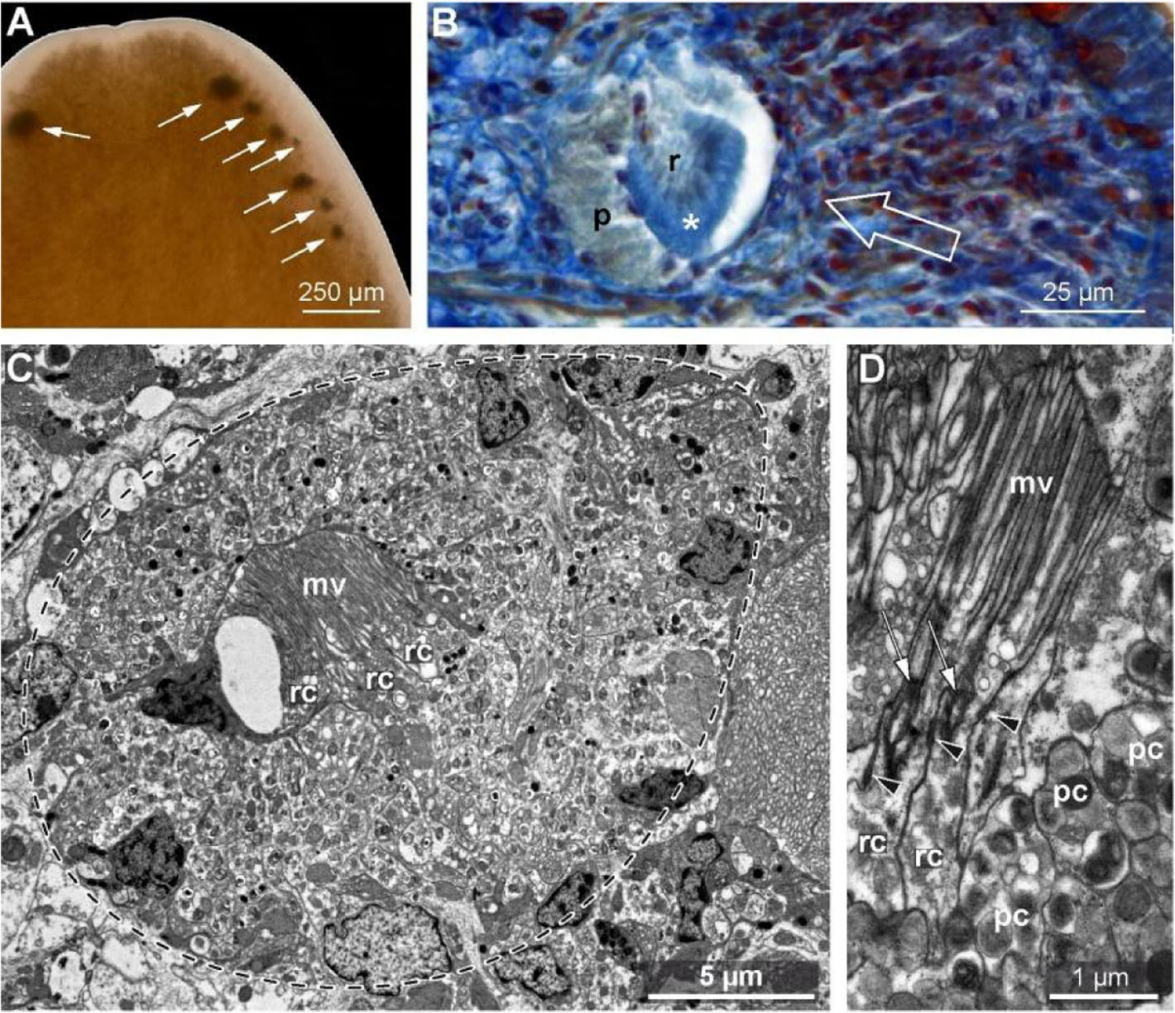
Sensory cells under different stainings exemplified for photoreceptors. **A-D***Lineus viridis* (Nemertea), anterior end. **A** Bright-field microscopy. Note that shading pigment marks the photoreceptors (*arrows*). **B** Azan staining of 5 μm thick section, bright-field. Receptor cells (*r*) are partly surrounded by shading pigment cells (*p*). *Asterisk* marks receptive region that can easily be identified by its homogenous staining and its apical position; neither single receptor cells nor the structure of the receptive region can be identified. *Arrow* marks direction of incident light. **C** Transmission electron microscopy, sagittal section; eye outline marked by *dashed line*. Shading pigment cells contain numerous vesicles with electron grey to electron dark content. **D** Detail of the receptor cells (*rc*) that consists of a dendritic process with numerous apical microvilli (*mv*) and a single cilium (*arrow*). Both, cilium and microvilli are constitutive for a certain class of photoreceptor cells. Note differing electron density of the vesicle content shading pigment cells (*pc*). *Arrow heads* mark adhaerens junctions indicative for epithelial sensory cells

Epithelial receptor cells are modified epithelial cells with an axon (primary sensory cells) or without an axon (secondary sensory cells). Epithelial receptor cells always face the exterior medium, the gut lumen, body cavities, or smaller compartments such as the optical cavity (Fig. 7B).

Modification generally enlarges the apical surface of epithelial receptors and causes branching, coiling, or ramification of the cilia, often along with altered axonemal and rootlet structures and/or elongation or branching of the microvilli (Fig. 7C). On the electron-microscopical level, they can be identified by apical adherence junctions linking them to neighboring cells (Fig. 7D). Under Nomarski contrast, they can easily be identified by their structure ([65] for olfactory cells). Silver impregnation methods that stain the axon allow detecting epithelial receptor cells if they are primary sensory cells. Certain epithelial receptor cells can be identified by antibody labelling against tubulin if the perceptive site is ciliary, or against neurotransmitters. Isolated secondary sensory cells can barely be discriminated from other epithelial cells since they lack an axon. Providing evidence for neurotransmitters is therefore decisive for identifying them. This can either be done by antibody labelling if the neurotransmitter is known [66]. Decisive identification of secondary sensory cells needs electron microscopy because 3D reconstruction allows identifying the postsynaptic processes penetrating the basal side of the secondary sensory cell and the neurotransmitter vesicles being located next to them [67].

Depending on the stimulus, receptor cells are classified as mechanoreceptors, chemoreceptors, photoreceptors, and others. Although the structure of the receptor cell is influenced by its specificity for a certain stimulus, such functional definitions pose problems on recognizing a member of a certain kind of receptor cell by its anatomy, since function and structure are not unambiguously correlated. Photoreceptors and chemoreceptors, for instance, possess enlarged surfaces such as modified microvilli [68, 69], so that this anatomical criterion cannot be used to discriminate between them. There are also receptor types that show differing anatomies, although their function is similar or identical. Mechanoreceptors are receptor neurons in the case of the muscle spindle or epithelial receptor cells in the case of the hair cells of the inner ear in vertebrates. Photoreceptors, finally, are secondary sensory cells in vertebrates, but primary sensory cells in several invertebrate taxa.

For most receptors, a combination of differential diagnosis and empirical comparative approaches are applied to unravel the potential function of a receptor if experimental approaches are not feasible. The structure of the transducer depends on its specificity for a certain stimulus, so that anatomy often allows inferring the function of receptor cells which are not experimentally accessible.

The problem of finding clearly specifiable spatio-structural recognition criteria for receptor cells reflects the general problem with specifying recognition criteria for functionally defined anatomical entities. Often, only physiological experiments can clearly identify a particular functionally defined anatomical entity. If such experiments cannot be conducted, morphologists are restricted to differential diagnostic and empirical comparative approaches coupled with conclusions based on analogy (Table 4).

**Table 4:** General diagnostic criteria of functional anatomical entities

***Method***	Recognition Criteria
differential diagnostic and empirical comparative approaches	*If structure X (in the position Y and combined with Z) thus far always had the function A, then structure X (in an identical position Y and combination Z) serves as recognition criterion for the functional unit A.*

#### Perception-based recognition criteria

**Fig. 8:**
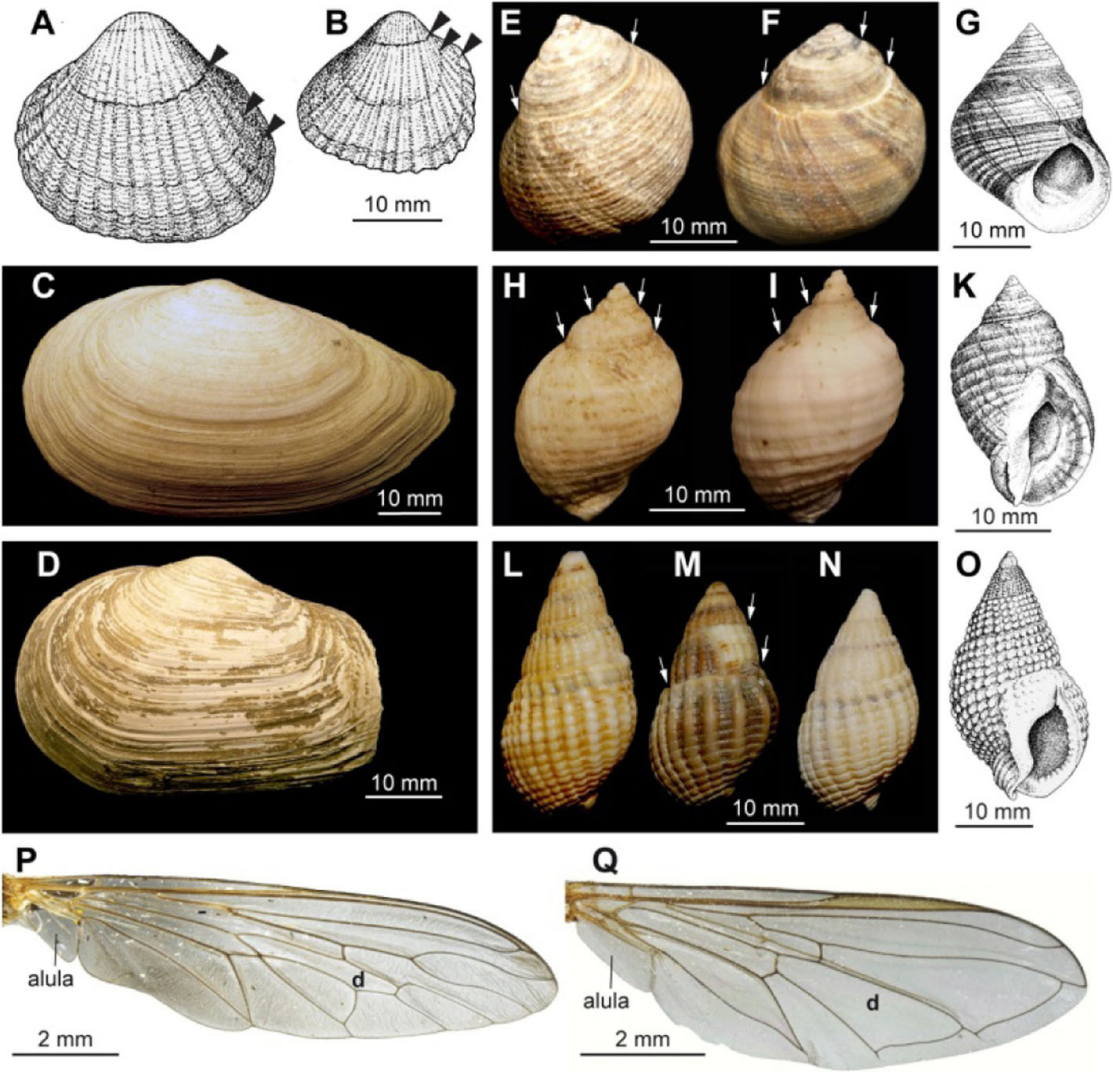
Lines and curves as diagnostic characters. **A**, **B** Shell outline differs between *Cerastoderma edule* (**A**) and *Cerastoderma glaucum* (**B**). Size differences refer to adult shells, *arrow heads* mark growth lines. **C**, **D** Truncate posterior end of the shell of *Mya truncata* (**D**) allows discriminating this species from its next relative, *Mya arenaria* (**C**). **E**-**O** Coiling of the gastropod shells causes sutures (*arrows*). Ridges, whorls, and outline a species-specific, despite difference in coloration. **E-G***Littorina littorea*, dorsal view (**E**, **F**), drawing of ventral view (**G**). **H-K***Nucella lapillus*, dorsal view (**H**, **I**), drawing of ventral view (**K**). **L-O***Nassarius reticulatus*, dorsal view (**L-N**), drawing of ventral view (**O**). Note intraspecific variation in color, ridges, and curviness in the shown gastropod shells. **P**, **Q** Wing veins in Diptera (Hexapoda), *d* exemplifies one homologous field. **P***Philonicus albiceps* (Asilidae, Diptera). **Q***Melanostoma mellinum* (Syrphidae, Diptera). **A**, **B**, **G**, **K**, **O** modified from Hayward and Ryland [70]

Iconic representational artifacts are often used in anatomy as the primary source of representation for communicating recognition criteria, as recognition criteria are often easier to convey in the form of visual examples than in the form of Aristotelian definitions and are essential for the training of diagnostic competence. Perception-based recognition criteria are also often used in taxonomic treatments through annotated images (Figs. 1, 2, 3, 4, 5, 6, 7 and 8).

By utilizing their natural relation of resemblance, perception-based recognition criteria demonstrate rather than denote the content to be communicated. In the following, we provide three examples where perception-based criteria are arguably more useful than Aristotelian definitions—in fact, Aristotelian definitions are not capable of efficiently communicating the diagnostic information.

##### Spatio-structural qualities

Many recognition criteria refer to specific spatio-structural qualities of anatomical entities. When having to define a term that refers to a specific spatio-structural quality, for instance, ‘mushroom-shaped’ or ‘ovoid’, we could try to textually define and describe the semantic conceptual content of the respective term, but this is often rather cumbersome and less comprehensible than simply visualizing its meaning through one or several exemplars—a picture is worth a thousand words. Anyone who has had to identify a species of a taxon that they were not familiar with using text-based keys knows how difficult it can be to make decisions based on textual criteria for shapes, colors, and relative positions, as opposed to using exemplary images.

**Fig. 9:**
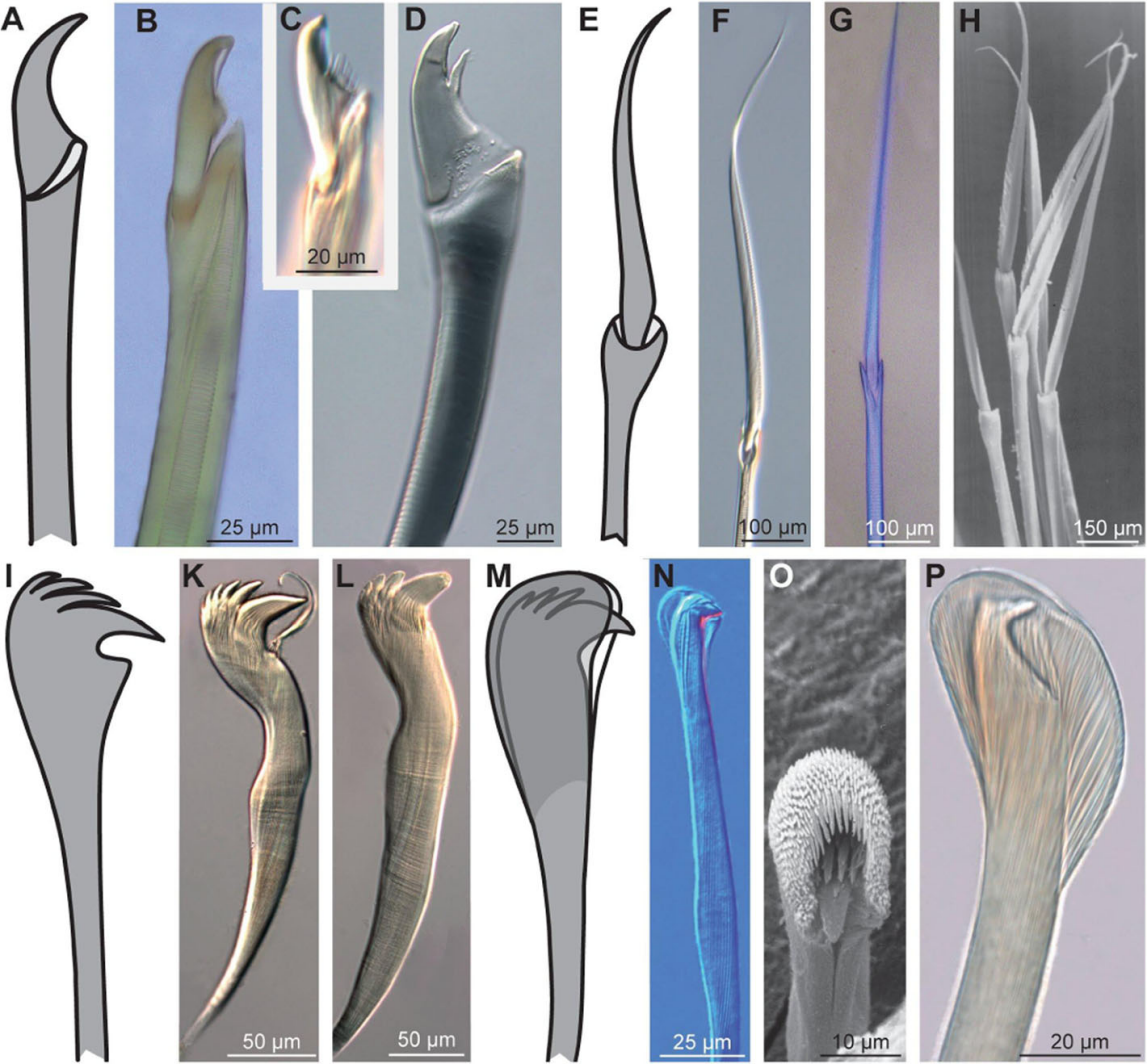
Chaetae in marine Annelida. **A-H** Schemes of falciger (**A**) and spiniger (**E**) compound chaeta and actual structure in different species. **B-D** Falcate compound chaetae under Nomarski contrast in *Perinereis cultrifera* (**B**), *Platynereis dumerilii* (**C**), *Stenelais boa* (**D**). **F-H***Spiniger* compound chaetae in *Platynereis dumerilii* (Nomarski contrast, **F**), *Perinereis cultrifera* (bright-field plus chitin autofluorescence, **G**), *Nereis diversicolor* (SEM, **H**). **I-P** Schemes of hooked chaeta (**I**) and hooded hook (**M**) and actual structure in different species. **K**, **L** Hooked chaetae in *Petaloproctus terricola* at different ages (L older than K) (Nomarski contrast). **N-P** Hooded hooks in *Dasybranchus caducus* under Nomarski contrast (**N**) and the SEM (**O**), *Lumbrineris tetraura* (Nomarski contrast, **P**)

Marine annelids, for instance, are generally identified on the species level according to the structure and arrangement of chaetae [71, 72]. These chaetae are extracellular structures that are formed by a single cell, the chaetoblast. Modification of the apical microvilli pattern of the chaetoblast during formation of the chaetae results in taxon-specific types of chaetae, which are discriminated by additional attributes like ‘hooked’, ‘falcate’, ‘falciger’, ‘winged’, ‘compound’ and others (Fig. 9). Due to the species-specificity of chaetae, these attributes are often insufficiently defined, so that pictures are used to provide an impression on what each attribute means.

Identification, in general, becomes significantly easier if keys provide images to relate a structure, an area, or a particular pattern to a certain term. The structure, area, or pattern is not defined semantically, but only by providing one or several images, sometimes in the form of schematic drawings that abstract the structure or pattern and its position. Actually, text in identification keys and taxonomic descriptions can be understood to be limited to the purpose of serving as figure legends, only aiding the understanding and accessibility of perception-based recognition criteria. The images define the respective term based on an ostensive definition, communicating the meaning of the term by pointing to one or more exemplars, or in the case of drawings and schemas sometimes also to a somewhat abstracted type.

If a term describes a particular outward appearance of an anatomical entity, for instance its shape or form, its particular relative position, or the particular distribution pattern of a spatially scattered group of entities, its ontological definition and recognition criteria coincides. In such cases, it would make sense to define the term only ostensively through exemplary images and abandon Aristotelian definitions altogether.

##### Training diagnostic competence

When teaching zoology and medical science, the students have to see a lot of structures prior to being able to identify them in a new, thus far unknown organism. Typical examples are found in basic courses, when we explain the function of, for instance, a nucleus, describe its general structure, and even provide a definition including textual recognition criteria for identifying a nucleus (see 4.1.1), but then we will show different pictures of nuclei as examples. It is not easy to explain without showing examples, what ‘coarse inner material’ or ‘electron-dense clusters’ actually means. Although textual recognition criteria for ‘nucleus’ exist, we use visual exemplars to get the students familiar with variation and train their ability to identify nuclei. The competence to identify an anatomical structure will grow with the number of different exemplars shown and will establish the idea of a type in the form of a generalized cognitive representation of its outer appearance.

Diagnostic competence is of major relevance in various disciplines, including anatomy and medicine, particularly pathology. Although textual recognition criteria for diseases do exist, their individual expression in patients varies to a degree that requires visual, audio, and haptic training to gain familiarity with the possible variation-space among diagnostic characters. The reliability of diagnoses in medicine, therefore, grows with experience and thus with the number of examples seen and studied.

##### Terms that divide a continuum into (arbitrary) discrete classes

In anatomy, we are often dealing with a continuum of very similar forms, shapes, colors, and spatio-structural patterns. There is a continuum of degrees of concavity/invagination of a surface or a continuum of degrees of density of hair. Traditionally, such continua are partitioned into discrete classes, with each class having its own associated term. We then use these terms to indicate where in the continuum the anatomical entity we are currently looking at is located.

When, for instance, looking at a boundary line of some anatomical structure, we might want to express that this line is curved. Curviness, however, comes in degrees and with a direction (concave or convex). Any curved line is thus positioned within the corresponding continuum of variants of curviness (Fig. 1). Instead of coining various terms to specify, which degree of curviness applies to the given boundary line, and provide textual definitions for each of them, we can use exemplary images, assign to each such image a URI and refer to the respective URI when describing the curviness of a particular boundary line. The exemplary images and their corresponding URIs can be ordered into a linear sequence (see above) or positioned within an anatomical space and then positioned relative to each other within the continuum. Adding further exemplars in the future, because one realizes that the resolution is not fine-grained enough, would also be straight forward. In practice, when describing the curviness of a boundary line, a morphologist would not have to search for the correct term anymore, but instead look at the ordered sequence of exemplary images and choose the image that best matches with the given boundary line [73] or the two images in between which the boundary line can be located as ‘more curvy than’ and ‘less curvy than’. The same approach can also be used for providing perception-based recognition criteria for the identification of different states of a linear process, as for instance neurulation (see Fig. 2B).

#### Mental representation of semantic and perceptual knowledge

Language is an essential tool for thinking and we mentally represent knowledge in a propositional form, i.e., as statements. This points to the question of *how* we process and store contents in our brain, which refers to the format that our knowledge can take when we mentally represent experiences (see *imagery debate* [74]). We can distinguish different types of contents, for example propositional and visual contents. A given content could for example be conveyed in oral English or in written English. Similarly, visual contents could be described using words or depicted in an image. Experiments about mental imagery indicate that we store visual contents in depictive formats [74] that are useful for memory as they allow the brain to store visual information more efficiently without first having to translate it into propositional knowledge. Knowledge which is implicitly contained in an image can be recovered retrospectively, although it was not explicitly considered at the time of encoding (i.e., mentally storing the image) [74]. This is also in line with the experience that each of us has already had that we often have to visualize things as a mental image to be able to answer questions about them (e.g., What color has the door to your apartment? What shape are a cat’s ears?). These findings in cognitive science point to the importance of depictively coded cognitive representations of sensory experiences that function as low-level mental representations. Our brain can employ this knowledge and relate it to propositional language when need be.

#### Concepts of grouping in addition to Essentialistic classes

Our examples from anatomy and the findings in cognitive science show the importance of recognition criteria and that essentialistic classes do not cover all types of groupings morphologists are dealing with. Biological entities are the product of evolution and exhibit a considerable degree of variation and diversity. When we define an anatomical entity by a set of predicates, we must exclude many individual entities that lack one or more defining predicates. This problem relates to what Nietzsche addressed when he stated that “only something which has no history can be defined” [75]. If the Aristotelian definition is too broad, the essentialistic class includes too many variants and becomes meaningless, and if the definition is too narrow, the number of classes for completely representing biological diversity would be overwhelming. Therefore, when used as defining properties of a class, the recognition criteria themselves do not necessarily define an essentialistic class.

If we apply the different methods to visualize neurons to different representatives of a species, the artifacts caused by the method will likely vary according to the specific function of the neurons, their interaction with other neurons, and the age of the organism. In other words, although ‘neuron’ is ontologically defined as an essentialistic class, instances of ‘neuron’ cannot be consistently identified with the same methods. Therefore, the class that is defined by the set of recognition criteria of ‘neuron’ *cannotbe represented as an essentialistic class*. Another concept of grouping is required.

Comparative morphologists are routinely confronted with an even more complex situation if they, for instance, want to identify photoreceptor cells in representatives of different species. Although a photoreceptor cell can be ontologically defined as an essentialistic class with a specific set of essential properties,^4^ no visualization method will be able to unravel all these properties at once. Each method applied will visualize certain particulars of the photoreceptors, which will likely not be identical across the different species. Depending on the life history stage of the studied organism, its species membership and the method used, each artifact will likely visualize a different subset of those properties that define the essentialistic class ‘photoreceptor’.

Among the defining properties of an essentialistic class ‘photoreceptor cell’ are functional dispositions. Functional dispositions, however, cannot be recognized without conducting physiological experiments, which are not only laborious but also invasive and may not be applicable in multi-species examinations. Instead, the presence of structural components or of special molecules in the signal transduction chain are interpreted as indices for the functional disposition. This interpretation is based on analogy and, thus, on abductive reasoning.

### The type concept in biology

In addition to essentialistic classes, other concepts of grouping have been applied by morphologists that are often based on some *type*. Although the *type* concept has been controversially discussed in the past [76–87], it has always been an influential concept in biology.

The *type* concept in biology is heterogeneous and represents a whole family of different concepts, which should be clearly distinguished. Farber [88] distinguished three different basic accounts of type in biology: a morphological, a classification, and a collection type concept.

#### The morphological type concept: a basic plan of organization

Until the mid-nineteenth century, illustrations of plants and animals were not intended to realistically represent anatomy. Illustrations had to transcend the species or genus, depicting the general and real *type* that was considered to be hidden. Many naturalists regarded this real *type* to be *truer to nature* and thus more real than any particular specimen [89]. The resulting illustrations represented *idealizations* (*reasoned image* sensu [89]). When conceptualizing taxonomic characters, naturalists had to ignore all irrelevant details and attempted to identify and separate essential from accidental traits to delimitate species and discover their *essential nature*. To reveal the universal in the particular, naturalists attempted to identify the essential or typical of a kind by synthesizing experience. Through comparing, selecting, judging, generalizing, and perfecting, they wanted to eliminate not only the errors that result from the deficiencies of human sensation, but also all idiosyncrasies found in individual specimens. They attempted to generate the most typical model of a species or genus, the *type*, which had to truly stand for and represent a real kind. This way they wanted to tame variability to adjust it to their essentialist metaphysical beliefs.

Daubenton and Cuvier believed in the existence of basic plans of organization for different types of anatomical structures that apply to various groups of organisms. Some of these plans were thought to be restricted to a single taxonomic level, while others to several or all levels. Cuvier considered them to be prototypes or archetypes that are prior to the things and operate like molds creating anatomical forms [90].

##### The morphological type concept in the twenty-first century

The morphological *type* concept survived for instance in textbook representations of the vertebrate brain. Every biologist knows illustrations of a typical fish, amphibian, bird, and mammalian brain, which show differing dominances of certain brain areas across vertebrate groups. These *types* neither represent brains of representatives of living species nor ancestral brain structures inferred from phylogenetic hypotheses. The typological approach is especially evident in the case of the *fish-brain* which shows characteristics of the highly derived teleost fishes.

Functional morphologists interpreted morphological *types* to represent fixed patterns of organization while, for instance *Naturphilosophen* such as Carus, interpreted them as ideal forms or archetypes based on a priori principles. Very few of these ideas have survived until today. A remarkable example is the dipleurula larva, which is a hypothetical larva of eleutherozoan echinoderms that still influence hypotheses on the evolution of life history strategies in metazoa [91].

Some biomedical ontologies have been developed based on the assumption of *canonical anatomy* [92], which can be considered to represent a notion of the morphological *type* concept. Canonical anatomy “comprises the synthesis of generalizations based on anatomical observations that describe idealized anatomy (structure)”, as opposed to *instantiated anatomy*, which “comprises anatomical data pertaining to instances (i.e., individuals) of organisms and their parts” ([92] p. 480; see also [10, 93]). The notion of canonical anatomy is important in medical contexts [94, 95], in which any deviation of a defined ‘normal’ condition or state is important for therapeutic purposes. It is also important whenever mutant forms are compared to a predefined wild type condition, as it is frequently done in genetic studies [96–98].

In a certain sense, kind terms in anatomy ontologies represent concepts, in which the ontological definitions are stripped down to contain only attributes that are universally applicable to all instances of the corresponding class. This generalization, however, cannot be compared to the process of inferring a real *type* that is based on some essentialist metaphysical beliefs or a prototype that is based on Platonic idealism. It rather represents a process of making non-idealized generalizations based on empirical knowledge.

##### Assessing the morphological type concept

Irrespective of its conceptual diversity, the morphological *type* concept always involves *abstraction* from individual organisms or populations and never is applied to an individual entity [99]. Epistemologically, it represents the result of a generalization from a group of particulars to some underlying universal and can provide the ontological definition for a morphological *type*, as well as its recognition criteria. However, to be able to generalize defining properties over a class, class membership must be known first. It is not suited for classifying particulars into meaningful groups, but rather presupposes the existence of a group and knowledge of a sufficient number of instances to be able to infer the universal that underlies all particular members of this group.

#### The classification type concept: a taxonomic model

The classification *type* concept has been used by naturalists such as Leclerce, de Buffon, Linnaeus, and Brisson primarily for nomenclatural purposes, to reduce repetition of character descriptions in taxonomy. This classification *type* concept does not provide any means for grouping anatomical entities into general types and *does not represent an ordering concept at all*.

While the reference to a taxon when referring to a structure (e.g., tetrapod limb) represents an abstraction from individual organisms to some universal entity, and thus qualifies as an application of the morphological *type* concept, we believe that in many cases it is closer related to the classification *type* concept and owed to the general problems involved when attempting to unambiguously translate perceptual non-conceptual contents related to shape and topological patterns into textual representational artifacts.

##### Assessing the classification type concept

Although there is no direct need for the classification *type* concept in anatomy, a taxon-specific attribute may be added to a term that refers to some type of anatomical entity to further specify it. The usefulness of such attributes results from restricting the number of applicable terms and the implicit contents that are communicated through the reference to a specific taxon. Taxon-specific attributes always refer to structural components that are exclusive in a given taxon for the structure in question. A *tetrapod-limb* is always pentadactyl without any exception. In other words, taxon-specific attributes are sometimes used as a sort of abbreviation for more detailed descriptions.

#### The collection type concept: a name-carrying specimen

*Type* specimens are individual specimens, described by the nomenclatural author of a species, and are primarily conceived as *models* and *name carriers*, which, ideally, should also possess the distinctive (essential; *differentia*) properties of the species. They also serve as *reference material* for later re-examinations.

##### Assessing the collection type concept

The collection *type* concept refers to particular specimens and is used for comparison and as a model for determining species membership and establishing new species and *does not represent an ordering concept* (it is “a method rather than a concept” [99]; p. 397). In anatomy, we make ample use of particular models as reference material for anatomical terms, especially in the context of terms for specific topological patterns and shapes (see *Spatio-structural qualities*) and for partitioning a continuum into discrete classes (see *Terms that divide a continuum into (arbitrary) discrete classes*). Therefore, we need to apply some notion of the collection *type* concept in anatomical research for naming anatomical entities based on perceptual non-conceptual contents.

In anatomy ontologies, the notion of a collection *type* concept has not been used yet, as the focus for developing ontologies has been on guaranteeing computer-parsability and on providing inferential lexical competence to users by defining the ontological nature of real entities [22] rather than on providing referential lexical competence through recognition criteria for securing the correct application of a term.

#### The role of the type concept in biology

We believe that besides the three distinct *type* concepts discussed above, there is also a more colloquial use of the term *type* in biology. Biologists frequently refer to a notion of *type* that is relatively vague and that takes the function of a *container concept* for all those cases in which the essentialistic class concept is inapplicable for organizing particular biological entities into a group.

Our examples above demonstrate that some cognitive representations of real entities exhibit a gap between their ontological definition and their empirical recognition criteria, resulting in groupings that are based on some sort of *type*. *This gap represents a conceptual inconsistency between the kinds of grouping in ontologies versus those used in scientific empirical research practice*.

Examples involve theoretical entities in biology, as for instance homologues, biological species, or monophyletic taxa, which are *types* of entities whose existence cannot be identified directly, but only indirectly, mediated through diagnostic characters [20]. They are generally *identified* by comparing patterns of shared similarity in the context of phylogenetic analyses [7, 100]. Their recognition criteria thus lack clearly defined necessary and sufficient criteria for class membership.

The conceptual inconsistency can also be found in the examples that we discussed further above, when comparing the ontological definition of, for instance, a cell nucleus and its method-dependent recognition criteria (Table 1, Fig. 3). The gap between ontological definition and empirical recognition is even more evident in those cases in which an anatomical entity is primarily defined by its function, e.g., receptor cells.

For some universals, it is difficult to capture their ontological nature using words, because our knowledge of them is limited to spatial and temporal properties that cannot be easily described in text. In other cases, the ontological definition does not allow the direct deduction of any empirically relevant recognition criteria, because the defining properties belong to entities that are too small to be visible to the naked eye or they are not directly observable at all, like for instance historical properties (e.g., homologues, monophyla), or the definition refers to a specific type of process which cannot be observed due to specific circumstances (e.g., functional structures in dead specimens). In such cases, morphologists often cannot apply essentialistic class concepts for the recognition of instances of a class but must resort to some vague notion of *type*.

This vague notion of *type* is usually applied for (1) purely descriptive purposes, (2) as an approximation and first step for discovering and demarcating essentialistic classes, or (3) as a diagnostic concept for recognizing class membership to an essentialistic class. This might also explain why essentialistic classes usually take prominent roles in biological theories, whereas some notion of *type* is predominantly applied in actual empirical research practice, diagnostics, or in education.

### Cluster analysis

All methods for recognizing and demarcating *types* can be associated with Ereshefsky’s *cluster analysis* [101], in which members of a group must share a cluster of similar traits, none of which is individually necessary and jointly sufficient. Many different types of cluster approaches exist, usually differing in the degree of similarity required for class membership and in the relationship between similarity and the theoretical ontological status of its instance members.

Most of the grouping methods refer to the notion of natural kinds rather than types of anatomical entities. According to Whewell’s method of *type* [102], a natural group of organisms epistemologically represents a class that is not determined by simply using a definition referring to a set of individually necessary and jointly sufficient properties but, instead, according to *total resemblance* in reference to a group-specific *type*, which also allows determining group membership “by characters which cannot be expressed in words” ([102] vol 1, p. 493). Groups created according to Whewell’s method of *type* are defined ostensively. Ostensive definitions refer to groups of things by pointing to one or more *exemplars* (i.e., Whewell’s *types*). However, Whewell [102] also points out that the method of *type* has been employed in combination with what he calls the idea of *natural affinities*, and that the impressive success of biological taxonomy is owed to the combined application of the method of *type* and the idea of natural affinity (today, we would call the latter phylogenetic homology).

Winsor’ *method of exemplars* [99], on the other hand, requires the identification of a typical member of a group and subsequent membership determination by comparison to that *type*. The method uses a *described form* (of specimens, species, or taxa) as an exemplar for comparison and for the evaluation of overall resemblance. Group membership is determined by passing a certain percentage of overall resemblance.

Wittgenstein’s concept of *family resemblance* [56, 99, 101, 103] goes a step further than the two methods discussed above and allows grouping of organisms through a *series of overlapping similarities*, with no specific set of features being necessarily common to all members of the group. Thus, starting with identifying anatomical entities that closely resemble a given exemplar entity, one can continue to use these closely resembling entities for the next similarity search, and so on. As a result, one might identify anatomical entities as belonging to the group although they have nothing in common with the initial exemplar. Whenever we relate different conditions to one another as belonging to the same continuum of forms, as for instance different degrees of density of hair (see 4.2.3), we apply the concept of family resemblance. It provides the basis for grouping groups that for instance have been established based on Whewell’s method of *type* or Winsor’s method of exemplars.

According to Ereshefsky [101], essentialistic classes follow a conjunctive approach, requiring each member of a class to possess all defining properties, and family resemblance is a disjunctive approach, requiring a disjunction of traits for membership determination. All other cluster approaches to classification, among which Ereshefsky also counts the notion of overall similarity that requires the members to share a number of similar traits (i.e., pheneticism), are conjunctive and can be located between Wittgenstein’s disjunctive family resemblance and essentialism [101].

### Cluster classes

Although method-dependent recognition criteria can contain universal statements that specify necessary properties, these universal statements usually do not specify properties that are exclusive to instances of the defined class. In other words, recognition criteria often cannot be represented as a set of universal statements. Instead, recognition criteria often represent contingent statements and do not define essentialistic classes, but cluster classes. In cluster classes, class membership is determined by a *minimum quorum* (Table 5) [56]. An individual instance of a cluster class does not have to possess all defining properties. *Necessary* is that some of them apply, *sufficient* is a specified percentage threshold or a number of properties, which is the *minimum quorum*. Consequently, like in essentialistic classes, class membership is either *yes* or *no*. However, contrary to the Aristotelian definitions of essentialistic classes, the typical or prototypical definitions of cluster classes do not (exclusively) represent universal statements but contain (some) contingent statements.

**Table 5:** A table of six particulars (A-F) together with some of their properties (1–5) and their classification according to different concepts of grouping (based on [56])

*instances (particulars)*	A	B	C	D	E	F
*properties 1*	x	–	x	x	x	–
*properties 2*	x	x	x	x	–	–
*properties 3*	x	–	x	x	x	–
*properties 4*	x	x	x	–	x	x
*properties 5*	x	–	x	–	x	–
*essentialistic class membership*	yes	no	yes	no	no	no
*cluster class membership (minimum quorum 3)*	yes	no	yes	yes	yes	no
*cluster class membership (minimum quorum 4)*	yes	no	yes	no	yes	no
*fuzzy set membership*	5/5	2/5	5/5	3/5	4/5	1/5

The cluster class concept is thus in many cases better suited to represent empirical diagnostic knowledge about recognition criteria than the essentialist class concept and takes on a pivotal role in anatomical research practice.

From the methods of clustering analysis discussed above, Whewell’s method of *types* may result in cluster classes (in which case Whewell’s method provides the recognition criteria for an underlying natural kind, Whewell’s natural affinities). With all properties of the exemplar functioning as defining properties, Winsor’s method of exemplars on the other hand *always* results in cluster classes.

### Fuzzy sets

Cluster classes can be seen as being defined in reference to some semantically defined exemplar, using textual representational artifacts for which a certain degree of sameness represents the requirement for class membership. But if we look at some of the text-based criteria more closely, we realize that they often use terms that rely on visual experience to be applicable. What does ‘coarse inner material’ or ‘electron-dense clusters’ actually mean? Clearly, *manyof these text-based criteria depend on perception-based criteria*, but neither essentialistic class concepts nor cluster class concepts can cover them, since none of them are perception-based.

In case no minimum quorum is specified, and group membership is not a matter of *yes* or *no* but, instead, a matter of degree, the group does not represent a class anymore, but a fuzzy set [104–106] (Table 5). A fuzzy set is a non-essentialistic concept of grouping that allows for a graded membership of its elements. In anatomy, fuzzy sets become important whenever only perception-based criteria for group membership are available. Having to provide a quantitative measure of the degree of sameness between a given instance and the defining properties is in many cases impossible, with the consequence that neither the essentialistic class concept nor the cluster class concept is applicable. Whereas fuzzy sets can be defined using perception-based criteria, essentialistic classes and cluster classes are always defined using text-based criteria. Perception-based fuzzy sets use ostensive definitions that typically rely on human capabilities of pattern recognition.

Recognition criteria of essentialistic classes such as ‘nucleus’ or ‘neuron’ characterize cluster classes that are defined using terminology that is difficult to comprehend for non-domain experts. The terms used in the recognition criteria, in turn, carry content that is perceptual and cannot be effectively communicated via textual representational artifacts. Respective terms thus characterize fuzzy sets.

Fuzzy sets can be seen as being defined in reference to some exemplars, which are defined using iconic representational artifacts. The boundary conditions of a sufficient degree of sameness cannot be specified in a quantitative way—hence *fuzzy*.

From the methods of cluster analysis discussed above, only Whewell’s method of *types* can result in fuzzy sets, as it is not restricted to textual representational artifacts. In Wittgenstein’s family resemblance, each exemplar is only an element in the chain of resemblance relations and applying it does not result in fuzzy sets. However, family resemblance can play a role when relating several fuzzy sets to a group that is meant to cover the different conditions one wants to distinguish within a given type of continuum, for instance, different degrees of density of hair.

In our experience, every conceptualization of a new type of anatomical entity starts as a fuzzy set, with the goal to define it essentialistically. We thus start with answering the *How does it look?* question prior to answering the *What is it?* question. Fuzzy set membership is recognized based on similarity, defined and communicated using exemplars, and conceptualized based on direct empirical comparison and thus empirical experience.

## Discussion

### Biomedical ontologies and FAIR eScience-compliant data and metadata standards

Ontologies have the purpose to increase the lexical competence of their users and play a central role in the establishment of FAIR [4] and eScience-compliant data and metadata standards [3, 14, 18, 36]. The provision of transparent meanings of concepts for humans and machines by textual or highly formalized ontological definitions improves or confirms the semantic inferential lexical competence [22] of a user and thus contributes to the *concept aspect* of a FAIR and eScience-compliant *terminology standard* (see Table 6). Many biomedical entities can be ontologically defined using Aristotelian definitions, grouping their instances as essentialistic classes, which have properties that enable easy reasoning over them. However, terms referring to spatial or temporal qualities (see *Spatio-structural qualities*) are often ostensively defined and grouped as fuzzy sets—a concept of grouping that is not used in ontologies and that possesses computationally less favorable properties.

**Table 6:** eScience-compliant data and metadata standards

Standard	*Guiding question*	*Requirements*	Solution
**Terminology**			
**Concept**	*What is the meaning of a concept? What do we know of the corresponding kind?*	*Must provide a human-readable and machine-actionable ontological definition and thus unambiguous meaning (*i.e.*, semantic value) to a term. Must contribute to a person’s semantic inferential competence.*	ontology terms, controlled vocabularies, Aristotelian definitions, essentialistic classes, cluster classes, fuzzy sets, exemplars
**Nomenclatural**	*Which words or symbols are used for referring to a specific kind?*	*Must provide an unambiguous link between term (*i.e.*, word/symbol) and concept, giving meaning to terms. Must contribute to a person’s output inferential competence.*	unique identifiers such as URIs, preferred labels, synonyms
**Diagnostic**	*How can I recognize and identify instances of a kind? What does an instance of a kind look like?*	*Must provide human-readable and machine-actionable empirical recognition criteria (*i.e.*, operational definitions) that enable identifying instances of a kind. Must contribute to a person’s naming and application referential competence.*	ontology terms, controlled vocabularies, Aristotelian definitions, essentialistic classes, cluster classes, fuzzy sets, exemplars
**Assertion**			
**Format**	*Which syntax and file format must be used?*	*Must provide a format and a syntax that enables machine- actionability and that guarantees the comparability of data and metadata. The standard also requires applications that can translate data and metadata into a human-readable version.*	RDF/OWL and a semantic data model, combined with tools for human-readability
**Content**	*Which information is relevant?*	*Must provide a basic categorization and classification of contents and corresponding schemata for documenting them.*	domain-specific semantic data model

The provision of unique identifiers for concepts in the form of URIs that allow unambiguous reference and reuse of concepts for humans and machines improves or confirms the output inferential lexical competence [22] of a user and thus contributes to the *nomenclatural aspect* of a FAIR and eScience-compliant *terminology standard*.

Ontologies should also be a resource for increasing and confirming the naming and the application referential lexical competence of their users. However, biomedical ontologies currently lack method-dependent recognition criteria and thus do not provide sufficient diagnostic information for reliably naming and identifying real instances of concepts. As a result, they do not contribute to the *diagnostic* and *operational aspect* of a FAIR and eScience-compliant *terminology standard* and thus considerably limit their practical applicability. The importance of the referential lexical competence becomes clear considering that anatomy is both a diagnostic science and a descriptive science. Anatomy’s diagnostic knowledge must be included in biomedical ontologies.

Method-dependent diagnostic knowledge is often represented through a combination of text-based and perception-based criteria. Covering recognition criteria is particularly important to application ontologies, but also to domain reference ontologies. Both should provide relevant diagnostic (operational) knowledge. Unfortunately, most biomedical ontologies still lack this diagnostic knowledge and contain purely ontological knowledge.

The specification of diagnostic knowledge in a biomedical ontology is straightforward if it is only provided in natural language together with annotated images. However, diagnostic knowledge should also be machine-actionable and respective method-dependent operational definitions must be provided in a formal language [49, 107]. A formal specification would have to distinguish between (i) methods used in sample preparation, (ii) methods for visualization, and (iii) a description of the relevant visible phenomena that result from the previous two steps.

Including diagnostic knowledge in ontologies would also substantially improve their value as educational resources. Moreover, since morphologists usually first describe patterns of similarity in appearance, before they investigate an underlying shared ontological nature, they require the necessary terminology to do so. This terminology cannot be comprehensively represented using essentialistic classes. Cluster classes are one way to generalize over a set of anatomical entities that show a significant degree of variability but nevertheless share some common patterns. Cluster classes can be defined in ontologies, for instance by using *existential quantifications or cardinality restrictions* in OWL2 (http://www.w3.org/TR/owl2-primer/).

Anatomical atlases define anatomical terms by providing visual examples as references and thus apply fuzzy set concepts. Unfortunately, because they are defined ostensively, fuzzy sets are not well suited for ontologies: they are semantically less explicit and neither necessary and sufficient properties nor a minimum quorum of required properties can be defined. Their contents are not defined textually and therefore cannot be translated directly into a formal syntax. However, ontologies can provide terms based on fuzzy sets and ostensive definitions by linking these terms to respective online resources, like for instance indexed media items in data repositories (for anatomical contents, e.g., Morph·D·Base). This way, biomedical ontologies could provide the required perceptual non-conceptual contents through representative reference images and atlas-like image tables. Ontologies could include these links in a standardized way and thereby provide all relevant diagnostic information necessary for identifying instances of the respective class (i.e., real referents of a term) as unambiguous as possible. By linking a URI to the label of a term and the same URI to an annotated exemplary image, the meaning is opened for discourse and the annotated image can be used for non-text-based computer analyses, for instance, adaptive algorithms for pattern recognition. By organizing these URIs into groups, biomedical ontologies could also model continua [73]. If each term explicitly specifies the type of definition it uses (i.e., Aristotelian, minimum quorum, ostensive), machines could even identify with which term which analyses can be used.

By incorporating concepts based on cluster classes and fuzzy sets, ontologies in general could significantly increase their contribution to establishing FAIR and eScience-compliant *terminology standards*. Since ontology terms can be used in RDF and OWL expressions for documenting ‘factual’ descriptions, they indirectly also contribute to the *format aspect* of a FAIR and eScience-compliant *assertion standard*. The *format aspect* requires, in addition to using RDF and OWL expressions, also the employment of semantic data models that guarantee a standardized way of representing similar types of data/metadata and human-readable versions of the information stored in the resulting semantic graphs. The semantic models, in turn, would contribute to the *content aspect* of a FAIR and eScience-compliant *assertion standard* that specifies which information is relevant for a given type of assertional statement.

## Conclusion

Biomedical ontologies must be applicable in diagnostic contexts as a knowledge source for researchers interested in identifying instances of a certain structural kind or software applications that facilitate diagnostic decision making and must therefore contain the relevant diagnostic knowledge. Whenever ontologies are used to link instances to each of their terms to form semantic knowledge graphs or for semantically annotating existing data or images, diagnostic knowledge is relevant. To guarantee comparability of diagnostic inferencing over different semantic knowledge graphs and annotated datasets, incorporating diagnostic knowledge should already be anticipated when developing the ontologies. Therefore, respective ontologies must be able to handle essentialistic class concepts alongside with cluster class concepts and fuzzy sets and differentiate between the ontological definition of a term and its method-dependent recognition criteria. Ideally, both ontological definition and method-dependent recognition criteria are formally represented in a machine-actionable format. Diagnostic knowledge could be extracted and used for keys to identify instances of structural kinds. If they are sufficiently detailed, they could even be utilized by adaptive algorithms for image processing and pattern recognition to facilitate automatic semantic annotation of media contents.

Existing biomedical ontologies usually lack diagnostic knowledge, and it needs a concerted effort to add the relevant knowledge to their terms. Adding and thus documenting diagnostic knowledge together with corresponding exemplary images, however, would be highly desirable considering its perceptual-heavy contents which are not sufficiently covered in the published literature. Currently, diagnostic knowledge is mostly carried by domain-experts who acquired it through gathering experience during years of comparative studies. Documenting their diagnostic knowledge would preserve it for future generations.

Using annotated exemplary images in biomedical ontologies also makes sense from the perspective of efficiency and economics, because understanding and interpreting annotated images is easier than reading and interpreting textual contents and can be processed significantly faster. This is also supported by experiments in cognitive sciences, which demonstrate that we mentally process and store visual information primarily as perceptual non-conceptual contents [74]. Texts, especially when describing spatio-structurally complex properties, can easily become unintelligible.

When looking at the relationship between recognition criteria and ontological definition and the role of perceptual non-conceptual contents in diagnosis, two things become obvious: (1) Most recognition criteria depend on a combination of cluster class concepts that rely on terms that refer to fuzzy sets. This explains why it is so difficult for non-domain-experts to understand anatomical descriptions and use anatomical data in their research. Providing all relevant diagnostic knowledge in respective ontologies, combined with annotated exemplary images that carry the relevant perceptual non-conceptual contents, would substantially increase the general usability of anatomical data. (2) Essentialistic classes play an important role in biological theories and hypotheses, whereas cluster class concepts and fuzzy sets are more important in the operational context of diagnosis. Thus, it is no surprise that the former attracted more attention in theoretical and methodological literature than the latter, as they had to be discussed alongside the justification of the respective theories or hypotheses. Since fuzzy sets and cluster classes have not been discussed in that detail in the biological community, biologists developed their own rather fuzzy conception of grouping that facilitated their diagnostic needs, the *type* concept. In many cases, reference to some notion of *type* can be replaced with either a cluster class or a fuzzy set concept, both of which can be elaborated on and made more specific than referring to a *type*, thereby improving the theoretical and conceptual basis of diagnostics in anatomy in particular and in the life sciences in general.

## Supplementary Information

**Additional file 1: S1 Glossary.** Definitions for terms marked with **↑** in the paper.

## Data Availability

Not applicable.

## Abbreviations

CLSM: Confocal Laser Scanning Microscopy
DL: Description logics
ECM: Extracellular Matrix
FAIR: Findable, Accessible, Interoperable, Reusable
OWL: Web Ontology Language
PATO: Phenotype Quality Ontology
RDF: Resource Description Framework
SEM: Section Electron Microscopy
URI: Uniform Resource Identifier

## Footnotes

1 Following Snodgrass [5], see also [6], we refer to the physical and structural properties of organisms as well as to the science of anatomy, with anatomical data being facts about the anatomy of organisms; the term ‘morphology’ we understand as interpretations of these facts within theories and hypotheses such as homology; however, we follow the common practice and call researchers of anatomy and researchers of morphology both ‘morphologists’.

2 We use “epistemological” here in the sense that it involves the application of methods and techniques conducted by the investigating researcher.

3 “But all science would be superfluous if the outward appearance and the essence of things directly coincided” [63], p. 592.

4 epithelial; connected to neighboring cells by adherens junctions; having an enlarged apical surface consisting of folded cell membranes; showing a retinal-opsin reaction upon being hit by a photon followed by a signal transduction cascade that finally changes the polarization of the axonemic membrane

## References

[1] Gray J, Hey T, Tansley S, Tolle K (2009). Jim Gray on eScience: A Transformed Scientific Method. The Fourth Paradigm: Data-Intensive Scientific Discoveries.

[2] Wang X, Gorlitsky R, Almeida JS (2005). From XML to RDF: how semantic web technologies will change the design of “omic” standards. Nat Biotechnol.

[3] Vogt L (2013). eScience and the need for data standards in the life sciences: in pursuit of objectivity rather than truth. Syst Biodivers.

[4] Wilkinson MD, Dumontier M, Aalbersberg IJ, Appleton G, Axton M, Baak A (2016). The FAIR guiding principles for scientific data management and stewardship. Sci Data.

[5] Snodgrass RE (1951). Anatomy and morphology. J N Y Entomol Soc.

[6] Richter S, Wirkner CS (2014). A research program for evolutionary morphology. J Zool Syst Evol Res.

[7] Vogt L, Bartolomaeus T, Giribet G (2010). The linguistic problem of morphology: structure versus homology and the standardization of morphological data. Cladistics..

[8] Stevens R, Goble CA, Bechhofer S (2000). Ontology-based knowledge representation for bioinformatics. Brief Bioinform.

[9] Bard J (2003). Ontologies: formalising biological knowledge for bioinformatics. BioEssays..

[10] Rosse C, Mejino JLV, Burger A, Davidson D, Baldock R (2007). The foundational model of anatomy ontology. Anatomy ontologies for bioinformatics: principles and practice.

[11] Smith B, Ashburner M, Rosse C, Bard J, Bug W, Ceusters W (2007). The OBO foundry: coordinated evolution of ontologies to support biomedical data integration. Nat Biotechnol.

[12] Vogt L. Spatio-structural granularity of biological material entities. BMC Bioinformatics. 2010;11(289) Available from: http://www.ncbi.nlm.nih.gov/pubmed/20509878. 10.1186/1471-2105-11-289 PMC3098069 20509878

[13] Vogt L (2008). Learning from Linnaeus: towards developing the foundation for a general structure concept for morphology. Zootaxa..

[14] Vogt L (2009). The future role of bio-ontologies for developing a general data standard in biology: chance and challenge for zoo-morphology. Zoomorphology..

[15] Dahdul WM, Balhoff JP, Engeman J, Grande T, Hilton EJ, Kothari C (2010). Evolutionary Characters, Phenotypes and Ontologies: Curating Data from the Systematic Biology Literature. PLoS ONE.

[16] Deans AR, Yoder MJ, Balhoff JP (2012). Time to change how we describe biodiversity. Trends Ecol Evol.

[17] Mungall CJ, Torniai C, Gkoutos GV, Lewis SE, Haendel MA (2012). Uberon, an integrative multi-species anatomy ontology. Genome Biol.

[18] Vogt L, Nickel M, Jenner RA, Deans AR (2013). The need for data standards in zoomorphology. J Morphol.

[19] Vogt L (2017). Assessing similarity: on homology, characters and the need for a semantic approach to non-evolutionary comparative homology. Cladistics..

[20] Vogt L (2018). Towards a semantic approach to numerical tree inference in phylogenetics. Cladistics..

[21] Vogt L, Baum R, Bhatty P, Köhler C, Meid S, Quast B (2019). SOCCOMAS: a FAIR web content management system that uses knowledge graphs and that is based on semantic programming. Database.

[22] Seppälä S, Ruttenberg A, Schreiber Y, Smith B (2016). Definitions in Ontologies. Cah Lexicol.

[23] Smith B, Floridi L (2003). Ontology. Blackwell guide to the philosophy of computing and information.

[24] Ramírez MJ, Coddington JA, Maddison WP, Midford PE, Prendini L, Miller J, Griswold CE, Hormiga G, Sierwald P, Scharff N, Benjamin SP, Wheeler WC (2007). Linking of digital images to phylogenetic data matrices using a morphological ontology. Syst Biol.

[25] Beck T, Morgan H, Blake A, Wells S, Hancock JM, Mallon A-M (2009). Practical application of ontologies to annotate and analyse large scale raw mouse phenotype data. BMC Bioinformatics..

[26] Balhoff JP, Dahdul WM, Kothari CR, Lapp H, Lundberg JG, Mabee P, Midford PE, Westerfield M, Vision TJ (2010). Phenex: ontological annotation of phenotypic diversity. PLoS One.

[27] Funk C, Baumgartner W, Garcia B, Roeder C, Bada M, Cohen KB (2014). Large-scale biomedical concept recognition: an evaluation of current automatic annotators and their parameters. BMC Bioinformatics..

[28] Dececchi TA, Balhoff JP, Lapp H, Mabee PM (2015). Toward synthesizing our knowledge of morphology: using ontologies and machine reasoning to extract presence/absence evolutionary phenotypes across studies. Syst Biol.

[29] Edmunds RC, Su B, Balhoff JP, Eames BF, Dahdul WM, Lapp H, Lundberg JG, Vision TJ, Dunham RA, Mabee PM, Westerfield M (2016). Phenoscape: identifying candidate genes for evolutionary phenotypes. Mol Biol Evol.

[30] Robinson PN, Mungall CJ, Haendel M (2015). Capturing phenotypes for precision medicine. Cold Spring Harb Mol Case Stud.

[31] Walter T, Shattuck DW, Baldock R, Bastin ME, Carpenter AE, Duce S, Ellenberg J, Fraser A, Hamilton N, Pieper S, Ragan MA, Schneider JE, Tomancak P, Hériché JK (2010). Visualization of image data from cells to organisms. Nat Methods.

[32] Dadzie A-S, Burger A (2005). Providing visualisation support for the analysis of anatomy ontology data. BMC Bioinformatics.

[33] Larson SD, Martone ME (2009). Ontologies for neuroscience: what are they and what are they good for?. Front Neuroinformatics.

[34] Ramírez MJ, Michalik P (2014). Calculating structural complexity in phylogenies using ancestral ontologies. Cladistics..

[35] Vogt L (2018). The logical basis for coding ontologically dependent characters. Cladistics..

[36] Vogt L (2011). Signs and terminology: science caught between language and perception. Bionomina..

[37] Smith B, Kusnierczyk W, Schober D, Ceusters W, Bodenreider O (2006). Towards a Reference Terminology for Ontology Research and Development in the Biomedical Domain. Proceedings of KR-MED 2006, Studies in Health Technology and Informatics, Vol 124.

[38] Smith B (1997). On substances, accidents and universals - in defence of a constituent ontology. Philos Pap.

[39] Smith B (2004). The logic of biological classification and the foundations of biomedical ontology. Spat Cogn Comput.

[40] Smith B, Varzi A, Vieu L (2004). Beyond Concepts: Ontology as Reality Representation. Proceedings of FOIS 2004 International Conference on Formal Ontology and Information Systems, Turin, 4–6 November 2004.

[41] Grice HP (1957). Meaning. Philos Rev.

[42] Hanna R (2005). Kant and nonconceptual content. Eur J Philos.

[43] Puget A, Mejino JLV, Detwiler LT, Franklin JD, Brinkley JF (2012). Spatial-symbolic query engine in anatomy. Methods Inf Med.

[44] Vernant J-P (1987). Mythos und Gesellschaft im alten Griechenland.

[45] Daston L, Galison P (1992). The image of objectivity. Representations..

[46] Heintz B (2000). Die Innenwelt der Mathematik: Zur Kultur und Praxis einer beweisenden Disziplin.

[47] Russell B (1905). On denoting. Mind..

[48] Schulz S, Stenzhorn H, Boekers M, Smith B (2009). Strengths and limitations of formal ontologies in the biomedical domain. Electron J Commun Inf Innov Health.

[49] Schulz S, Jansen L (2013). Formal ontologies in biomedical knowledge representation. IMIA Yearb Med Inform.

[50] Schulz S, Kumar A, Bittner T (2006). Biomedical ontologies: what part-of is and isn’t. J Biomed Inform.

[51] Gray H (1918). Anatomy of the human body.

[52] Mayr E, Bock WJ (2002). Classification and other ordering systems. J Zool Syst Evol Res.

[53] Audi R (1999). The Cambridge dictionary of philosophy.

[54] Mahner M, Bunge M (1997). Foundations of biophilosophy.

[55] Idrees SM, Alam MA, Agarwal P (2018). A study of big data and its challenges. Int J Inf Technol.

[56] Stamos DN (2005). Pre-Darwinian taxonomy and essentialism – a reply to Mary Winsor. Biol Philos.

[57] Smith B, Rosse C (2004). The role of foundational relations in the alignment of biomedical ontologies. Stud Health Technol Inform.

[58] Richter S, Loesel R, Purschke G, Schmidt-Rhaesa A, Scholtz G, Stach T, Vogt L, Wanninger A, Brenneis G, Döring C, Faller S, Fritsch M, Grobe P, Heuer CM, Kaul S, Møller OS, Müller CHG, Rieger V, Rothe BH, Stegner MEJ, Harzsch S (2010). Invertebrate neurophylogeny: suggested terms and definitions for a neuroanatomical glossary. Front Zool.

[59] Sowa JF (1984). Conceptual structures: information processing in mind and machine.

[60] Sowa JF, van Harmelen F, Lifschitz V, Porter B (2008). Conceptual graphs. Handbook of knowledge representation.

[61] Frege G (1891). Function und Begriff: Vortrag gehalten in der Sitzung vom 9. Januar 1891 der Jenaischen Gesellschaft für Medicin und Naturwissenschaft.

[62] Frege G (1892). Über Sinn und Bedeutung. Z Für Philos Philos Krit.

[63] Marx K, Engels F (1894). Capital: a critique of political economy; volume III: the process of capitalist production as a whole.

[64] Marconi D (1995). On the structure of lexical competence. Proc Aristot Soc.

[65] Anderson PAV, Hamilton KH (1987). Intracellular recordings from isolated salamander olfactory receptor neurons. Neuroscience..

[66] Boyer NP, Chen C, Koutalos Y. Preparation of living isolated vertebrate photoreceptor cells for fluorescence imaging. J Vis Exp. 2011;(52) Available from: http://www.jove.com/details.php?id=2789. 10.3791/2789 PMC3197052 21730941

[67] Budelmann B-U, Thies G (1977). Secondary sensory cells in the gravity receptor system of the statocyst of Octopus vulgaris. Cell Tissue Res.

[68] Purschke G (1997). Ultrastructure of nuchal organs in polychaetes (Annelida) – new results and review. Acta Zool.

[69] Purschke G, Bartolomaeus T, Purschke G (2005). Sense organs in polychaetes (Annelida). Morphology, molecules, evolution and phylogeny in Polychaeta and related taxa.

[70] Hayward PJ, Ryland JS (1995). Handbook of marine fauna of North-West Europe.

[71] Fauchald K (1977). The polychaete worms. Definitions and keys to the orders, families and genera. Nat Hist Mus Los Angel Cty Sci Ser.

[72] Hartmann-Schroeder G (1996). Annelida, Borstenwürmer, Polychaeta.

[73] Yoder MJ, Twidale MB, Thomas AK, Vogt L, Franz NM, Guo J, Thessen AE (2018). Taxonomy and the production of semantic phenotypes. Application of semantic Technology in Biodiversity Science Studies on the semantic web.

[74] Pearson J, Kosslyn SM (2015). The heterogeneity of mental representation: ending the imagery debate. Proc Natl Acad Sci.

[75] Nietzsche F, Ansell-Pearson K (1887). On the Genealogy of Morality.

[76] Mayr E, Meggers BJ (1959). Typological versus population thinking. Evolution and anthropology: a centennial appraisal.

[77] Mayr E, Meggers BJ (1959). Darwin and the evolutionary theory in biology. Evolution and anthropology: a centennial appraisal.

[78] Mayr E (1963). Animal species and evolution.

[79] Mayr E (1968). Theory of biological classification. Nature..

[80] Mayr E (1976). Evolution and the diversity of life: selected essays.

[81] Mayr E (1982). The growth of biological thought: diversity, evolution, and inheritance.

[82] Hull DL (1965). The effect of esstenialism on taxonomy: 2000 years of stasis. Br J Philos Sci.

[83] Balme D, Gotthelf A, Lennox JG (1987). Aristotle’s biology was not essentialist. Philosophical issues in Aristotle’s biology.

[84] Lennox JG (1987). Kinds, forms of kinds and the more and the less in Aristotle’s biology. Philosophical Issues in Aristotle’s Biology.

[85] Winsor MP (2006). The creation of the essentialism story: an exercise in metahistory. Hist Philos Life Sci.

[86] Walsh D (2006). Evolutionary essentialism. Br J Philos Sci.

[87] Wheeler QD, Valdecasas AG (2007). Taxonomy: myths and misconceptions. An Jardín Botánico Madr.

[88] Farber PL (1976). The type-concept in zoology during the first half of the nineteenth century. J Hist Biol.

[89] Daston L, Galison P (2007). Objectivity.

[90] Eigen EA (1997). Overcoming first impressions: Georges Cuvier’s types. J Hist Biol.

[91] Nielsen C (2013). Life cycle evolution: was the eumetazoan ancestor a holopelagic, planktotrophic gastraea?. BMC Evol Biol.

[92] Rosse C, Mejino JLV (2003). A reference ontology for biomedical informatics: the foundational model of anatomy. J Biomed Inform.

[93] Rosse C, Mejino JL, Modayur BR, Jakobovits R, Hinshaw KP, Brinkley JF (1998). Motivation and organizational principles for anatomical knowledge representation: the digital anatomist symbolic Knowledge Base. J Am Med Inform Assoc.

[94] Campbell K, Das A, Musen M (1994). A logical foundation for representation of clinical data. J Am Med Inform Assoc.

[95] Evans D, Cimino J, Hersh W, Huff S, Bell D (1994). Toward a medical-concept representation language. J Am Med Inform Assoc.

[96] Pal S, Liput M, Piques M, Ishihara H, Obata T, Martins M (2013). Diurnal changes of polysome loading track sucrose content in the rosette of wild-type Arabidopsis and the starchless pgm mutant. Plant Physiol.

[97] Liu Q, Zhou B, Ma W, Bawa B, Ma J, Wang W, Lang Y, Lyoo Y, Halpin RA, Lin X, Stockwell TB, Webby R, Wentworth DE, Richt JA (2014). Analysis of recombinant H7N9 wild-type and mutant viruses in pigs shows that the Q226L mutation in HA is important for transmission. J Virol.

[98] Jeong B, Wittmann C, Kato T, Park E (2015). Comparative metabolic flux analysis of an Ashbya gossypii wild type strain and a high riboflavin-producing mutant strain. J Biosci Bioeng.

[99] Winsor MP (2003). Non-essentialist methods in pre-Darwinian taxonomy. Biol Philos.

[100] Vogt L (2002). Testing and weighting characters. Org Divers Evol.

[101] Ereshefsky M (2001). The poverty of the Linnaean hierarchy - a philosophical study of biological taxonomy.

[102] Whewell W (1847). The Philosophy of the Inductive Sciences, Founded upon their History - Vol. 1.

[103] Wittgenstein L (1953). Philosophical investigations.

[104] Klaua D (1965). Über einen Ansatz zur mehrwertigen Mengenlehre. Monatsblatt Dtsch Akad Wiss Zu Berl.

[105] Zadeh LA (1965). Fuzzy stets. Inf Control.

[106] Zimmermann H-J (2001). Fuzzy set theory - and its applications.

[107] Schulz S, Karlsson D (2011). Records and situations. Integrating contextual aspects in clinical ontologies.

[108] Gupta A, Larson SD, Condit C, Gupta S, Fong L, Chen L, Hainaut J-L (2007). Toward an ontological database for subcellular neuroanatomy. Lecture notes in computer science (ER workshops 2007), LNCS 4802.

[109] Marr D (1982). Vision.

[110] Scholtz G (2014). Evolution of crabs – history and deconstruction of a prime example of convergence. Contrib Zool.

[111] Rieppel O (2007). The performance of morphological characters in broad-scale phylogenetic analyses. Biol J Linn Soc.

[112] Dahdul WM, Lundberg JG, Midford PE, Balhoff JP, Lapp H, Vision TJ, Haendel MA, Westerfield M, Mabee PM (2010). The teleost anatomy ontology: anatomical representation for the genomics age. Syst Biol.

[113] Yoder MJ, Mikó I, Seltmann KC, Bertone MA, Deans AR (2010). A gross anatomy ontology for Hymenoptera. PLoS One.

[114] Serna F, Bolton B, Mackay W (2011). On the morphology of Procryptocerus (Hymenoptera : Formicidae). Some comments and corrigenda. Zootaxa..

[115] Franz NM, Goldstein AM (2013). Phenotype ontologies: are homology relations central enough? A reply to Deans et al. Trends Ecol Evol.

[116] Franz NM (2014). Anatomy of a cladistic analysis. Cladistics..

[117] Linnaeus C (1735). Systema naturae sive Regna tria naturae. Systematice proposita per Classes, Ordines, Genera, & Species.

[118] Linnaeus C (1751). Philosophia botanica.

[119] Larson JL (1967). Linnaeus and the natural method. Isis..

[120] Ereshefsky M (1997). The evolution of the Linnaean hierarchy. Biol Philos.

[121] Liem KF, Bemis WE, Walker WF, Grande L (2001). Functional anatomy of vertebrates: an evolutionary perspective.

[122] Owen R (1848). On the archetype and homologies of the vertebrate skeleton.

[123] Camardi G, Owen R (2001). Morphology and Evolution. J Hist Biol.

[124] Rupke NA (1994). Richard Owen: Victorian naturalist.

[125] Amundson R (2005). The changing role of the embryo in evolutionary thought: roots of evo-devo.

[126] Brigandt I (2007). Typology now: homology and developmental constraints explain evolvability. Biol Philos.

[127] Rosse C (2012). The challenges of representing anatomical spatial relations. Methods Inf Med.

