# Supplementary material for: Anatomy and the type concept in biology show that ontologies must be adapted to the diagnostic needs of research
Source: J Biomed Semantics. 2022 Jun 27;13:18. doi: 10.1186/s13326-022-00268-2 (PMC9235205; doi:10.1186/s13326-022-00268-2)
Supplement: Supplementary file 1 — Additional file 1: S1 Glossary. Definitions for terms marked with ↑ in the paper. [file 13326_2022_268_MOESM1_ESM.pdf]

S1 Glossary

Definitions for terms marked with ↑ in the paper.

API↑

An application programming interface (API) is a computing interface that allows two applications to talk to each other. It functions like a messenger that delivers a particular request to the provider (e.g., asking a database for a specific type of data content) and then delivers the response of the provider. In other words, an API is a set of instructions and standards for accessing a (Web-based) software application. An API is released to the public so that other software developers can create products that utilize the services of this application.

Amazon.com released its API so that other software developers can easily access Amazon's product information. The GBIF API provides means to create, edit, update and search for information about datasets, organizations, networks, species, higher taxa, occurrence data and provides services to show maps of GBIF mobilized content on other sites (1).

Aristotelian definition↑

Aristotelian definitions are definitions for essentialistic classes↑ and their corresponding kind terms↑. Aristotelian definitions represent universal statements↑ and always consist of two parts, genus↑ and differentia↑, both of which specify essential properties that any instance↑ of the essentialistic class↑ must necessarily possess. Genus↑ specifies the parent class↑ of the essentialistic class↑ by referencing the corresponding kind term↑. The defining properties of the parent class↑ are inherited downstream to all its subclasses↑, including the essentialistic class↑. Differentia↑ specifies the essential properties of the essentialistic class↑ itself. Several Aristotelian definitions result in a hierarchical system of kind terms↑ with their corresponding essentialistic classes↑ and subclasses↑. This hierarchy can be represented as a tree and forms a taxonomy↑.

The differentia↑ part of an Aristotelian definition is required for distinguishing between all sister classes↑ (i.e. classes that share the same direct parent class) within a given hierarchical level in the taxonomy↑. The defining properties specified in genus↑ and differentia↑ are individually necessary and jointly sufficient for determining membership of an instance↑ to an essentialistic class↑.

Example for an Aristotelian definition of the kind term 'cell nucleus': Organelle which has as its direct parts a nuclear membrane and nuclear matrix. "Organelle" refers to the parent class and thus represents the genus part of the definition, whereas the remainder of the definition is the differentia part.

Assertional statement↑

Statements about particulars↑. In Description Logic, they are referred to as ABox expressions. (2–4).

Your friend, Bob, is American. The leg of this wasp specimen is red.

Big Data↑

Within science, Big Data is a field of science in which datasets too large or too complex to be dealt with by traditional data management and analysis tools, are analyzed and explored using new approaches. The key challenges of Big Data are capturing, storing, analyzing, searching, sharing, transferring, visualizing, querying, and updating large datasets as well as data privacy, metadata tracking and provenance (5). Big Data is related to eScience↑.

Class↑

A grouping concept for defining a general group↑. A class is always defined intensionally by a predicate (i.e. connotative definition). Class definitions are universal↑ or contingent statements↑ that carry semantic conceptual content↑. All instances↑ of a class possess the predicate that defines the class. We distinguish two types of classes: essentialistic classes↑ and cluster classes↑.(6,7) If a class possesses a real correlate, one can use class↑ for referring to the concept, kind↑ for referring to the real entity↑, and kind term↑ for referring to the word we use to refer to the class↑ and the kind↑. Following this notion, if classes have ontological correlates (i.e., kinds), classes are our cognitive representations↑ of these kinds↑.

The class of all red things. Cell nucleus defined as: Organelle which has as its direct parts a nuclear membrane and nuclear matrix.

Cluster Class†

A class† that is not defined by a set of universal statements† that are individually necessary and jointly sufficient. Instead, cluster classes (also called *polythetic classes* (8)) are defined by a set of contingent statements† so that class membership is determined by a minimum quorum (9). That means, a particular instance† of a cluster class does not have to possess all defining properties. *Necessary* is that some of them apply, *sufficient* is a specified percentage threshold or a number of properties, which is the minimum quorum. As a consequence, like in essentialistic classes†, membership to a cluster class is either *yes* or *no*.

Cognitive representation†

Cognitive representations are thoughts, perceptions, conceptions, ideas, and beliefs about a given real entity† (universal† or particular†) in the mind of the scientist. Everyone uses cognitive representations to create a mental model of their environment. We use two types of representational artifacts† when communicating with others about real entities† and their cognitive representations. The purpose of representational artifacts† is to induce cognitive representations in the receiver that resemble those held by the sender—we want to share the same cognitive representation when communicating about the same real entity†. (10,11)

Contingent Statement†

A statement that is true for some instances† of a specific universal† but not necessarily for every instance. (2–4)

Data repository†

A large database infrastructure that collects, manages, and stores data sets for data analysis, sharing and reporting. A data repository is also known as a data library or data archive.
NCBI GenBank is an example of a data repository for a sequence database.

Description logics (DL)†

DL is a family of formal knowledge representation languages and as such provides the logical formalism for ontologies and the Semantic Web. OWL† is based on DL. DLs are usually more expressive than propositional logic but less expressive than first-order logic. In DL, TBox expressions are distinguished from ABox expressions, with the former carrying universal statements† and the latter assertional statements†.

Diagnosis†

The act of identifying a particular† as an instance† of a kind† based on diagnostic empirical knowledge in the form of diagnostic characters (empirical recognition criteria†). In biology, diagnostic knowledge is often communicated through exemplars, images, or by doing (e.g. going together on a field trip and showing things) instead of textual recognition criteria. The competence to identify an anatomical structure grows with the number of different exemplars shown and establishes the idea of a type in the form of a generalized cognitive representation† of its outer appearance. Diagnostic competence is of major relevance in various disciplines, including anatomy and medicine, particularly pathology.

Dictionary†

A dictionary is a listing of words in a specific language, often arranged in an alphabetic order that provides lexicographical reference. Dictionaries may include for each word information on its definitions, usage, etymologies, pronunciations, translations etc.

Differentia†

Differentia, here, refers to the differentia part of an Aristotelian definition†.

(Empirical) recognition criteria†

Determine the cognitive representation† of an entity's appearance and thus provide answers to *How does it look?* questions that support scientists in their ability to successfully identify a real entity† as an instance† of a specific kind†. They represent the epistemological counterpart to ontological definitions† that provide answers to *What is it?* questions. Empirical recognition criteria specify diagnostic properties that are required for a scientist to successfully recognize and identify particular instances† of a given kind†. Sometimes, they are also referred to as operational definitions. Whereas ontological definitions†

carry semantic conceptual content↑ about the ontological nature of a kind↑, empirical recognition criteria are important for unambiguous reference↑ of instances↑ to a kind↑. Diagnostic properties can be communicated through text-based and perception-based recognition criteria.

eScience↑

A new independent approach to research, sometimes also referred to as e-Science, data exploration, data research, or eResearch, that complements the three established approaches to research, i.e. experimentation, theorizing, and computer modeling and simulations.(12,13) It applies to data-rich fields of empirical research and involves an intensive use of computers and algorithms for exploring and utilizing large amounts of data. eScience is "the application of computer technology to the undertaking of modern scientific investigation, including the preparation, experimentation, data collection, results dissemination, and long-term storage and accessibility of all materials generated through the scientific process. These may include data modeling and analysis, electronic/digitized laboratory notebooks, raw and fitted data sets, manuscript production and draft versions, pre-prints, and print and/or electronic publications." (14) eScience is related to Big Data↑.

Essentialistic class↑

A class↑ that is defined using an Aristotelian definition↑ is thus based on universal statements↑. Membership to an essentialistic class is determined in dependence on a set of properties that are individually *necessary* and jointly *sufficient*. As a consequence, membership of a given particular↑ to an essentialistic class is either *yes* or *no* (9). Classifying kinds↑ into essentialistic classes results in a hierarchical system of kind terms↑ that can be represented as a tree and forms a taxonomy↑ (15). Due to their clarity and simplicity, essentialistic classes are well suited for ontologies↑.

'Factual' description↑

A specific type of assertional statement↑ that summarizes the results of an observation of a particular↑. Everybody who reads a 'factual' description should receive the same or very similar cognitive representations↑ of a real entity. Only 'factual' descriptions qualify as data↑, as they represent observations recorded using words, numbers, and symbols. Terms used in 'factual' descriptions can be defined in ontologies↑ as universal↑ and terminological statements↑. (10,16)

FAIR↑

FAIR stands for Findable, Accessible, Interoperable, and Reusable and refers to the FAIR Guiding Principles for scientific data and metadata management and stewardship. Data and metadata must be stored and documented using formats and standards that allow humans and machines (see machine-actionable↑) alike to find, access, and reuse them and interoperate with other FAIR (meta)data. FAIR (meta)data use persistent identifiers↑, are indexed in a searchable resource, are openly, freely and universally retrievable by their identifiers, are documented using a language for knowledge representation with a vocabulary that follows the FAIR principles, use accessible data usage licenses, and come with detailed provenance information and meet domain-relevant community standards. [14,15]

Fuzzy set↑

A type of grouping of entities (i.e., elements) that does not specify a minimum quorum as in cluster classes↑ and for which membership is not a matter of *yes* or *no* as in cluster classes↑ and essentialistic classes↑ but, instead, a matter of degree. A fuzzy set is a non-essentialistic concept of grouping that allows for a graded membership of its elements (19–21). In science, fuzzy sets↑ are often used when dealing with uncertainty, incomplete knowledge, possibilities, and human cognition (21). Similar to cluster classes↑, fuzzy sets can be seen as being defined in reference to some exemplars. However, contrary to cluster classes↑, the exemplars are defined using iconic representational artifacts↑. The boundary conditions of a sufficient degree of sameness cannot be specified in a quantitative way—hence *fuzzy*.

General group↑

Ordering of entities within general groups in relation to group defining properties or by pointing out the members of the group. Grouping entities into general groups represents the most commonly used way to efficiently manage representational artifacts↑. We can distinguish sets↑ which are defined extensionally by pointing out their members and classes↑ which are defined intensionally by predicates as two basic grouping concepts.

Genus↑

Genus here refers to the genus part of an Aristotelian definition↑.

Iconic representational artifact↑

Iconic representational artifacts are, e.g., images, image stacks, 3D models, video and audio recordings of real entities↑. Such media items carry perceptual non-conceptual contents↑ (i.e., image-based or audio-based information) that do not denote but rather demonstrate (see *aesthetic nonconceptual content* (10)). The meaning contained in a media item is not arbitrary but rests on a natural relation of resemblance to the part of reality that it reproduces (*natural meaning* (22,23)).

Inferential lexical competence↑

One can distinguish inferential from referential lexical competence↑. The inferential lexical competence of a person depends on the person's knowledge about the meaning of a term and thus its semantic value↑. It relates to ontological definition↑ for a given term. Inferential lexical competence can be differentiated into *semantic inferential competence*, which relates to natural language and formal logical Aristotelian definitions↑ that contribute to the person's understanding of the intended meaning of a term and its use in inferential operations, and *output inferential competence*, which relates to the words and phrases (i.e., labels) used for referring to a specific concept, including synonyms but also the URI↑ used in an ontology↑ for identifying a class↑ or a relation. (4) Whereas inferential lexical competence↑ relies on a semantic system, referential lexical competence relies on a perceptual and motor system. Although these two systems are distinct, they both interact (4).

Instance↑

A particular↑ that instantiates a class↑.

I am an instance of the class parent.

Kind↑

A kind is a universal↑ object or process and thus refers to a real entity↑. Whereas class↑ is used for referring to the concept, kind↑ is used for referring to this real entity↑, and kind term↑ for referring to the word we use to refer to the class↑ and the kind↑. Following this notion, if classes have ontological correlates (i.e., kinds), classes are our cognitive representations↑ of these kinds↑ and kind terms↑ our textual representational artifacts↑.

Kind term↑

A kind term is the textual representational artifact↑ of a kind↑ (universal↑). (10,24)

'Particle accelerator' and 'homo sapiens' are examples for kind terms.

Knowledge base↑

A knowledge base is a set of ontology↑ classes↑ that are populated with empirical data. Empirical data are assertional statements↑ that are represented in the form of instance↑-based semantic graphs↑ (ABox expressions) (3). An ontology↑ contains universal statements↑ in the form of class↑-based semantic graphs↑ (TBox expressions). A knowledge base is constituted by a combination of TBox and ABox expressions (25).

Linear sequence↑

Ordering (positioning) of entities within a directed continuum, i.e. entity a is before entity b and after entity c.

A continuum of forms between two extremes, e.g., microvilli (evaginations) and crypts (invaginations). A continuum of values, e.g. the volume of a body. A continuum of forms or interactions as a time-ordered sequence.

Machine-actionable↑

Data and metadata that are structured in a formalized and consistent way so that machines (i.e. computers) can read and use them with algorithms that were programmed against this structure. Machine-actionability of data and metadata includes for instance the use of persistent identifiers↑ for data creators (e.g. ORCIDs), organizations and funding agencies, but also open accessibility of data for machines through a corresponding API↑, and basic semantics that allow algorithms to distinguish different

categories of information and apply rules to them. Machine-actionability in this sense goes beyond machine-readability which only requires data and metadata to be readable by a machine, i.e. data and metadata must be provided in a machine-readable format. Machine-readability does not necessarily require data and metadata to provide basic semantics for allowing algorithms to distinguish different categories of information contained in them.

Ontological definition↑

An ontological definition provides information about the ontological nature of a certain kind↑ of entity. It offers transparency regarding the semantic value↑ (i.e. meaning) of its associated kind term↑ by providing an answer to the *What is it?* question. It represents the ontological counterpart to the epistemological empirical recognition criteria↑ that also have been referred to as operational definitions and that provide answers to *How does it look?* questions. Ontological definitions specify defining properties that are required for a particular↑ to be an instance↑ of the corresponding kind↑. Whereas empirical recognition criteria↑ are important for unambiguous reference↑ of instances↑ to a kind↑, ontological definitions carry semantic conceptual content↑ about the ontological nature of a kind↑.

Ontology↑

Ontologies are dictionaries↑ that can be used for describing a certain reality. They consist of labeled classes↑ and relations between classes, both with clear definitions that are ideally created by experts through consensus and that are formulated in a highly formalized canonical syntax and standardized format with the goal to yield a lexical or taxonomic framework for knowledge representation (26). Each ontology class↑ and relation (also called property) possesses its own URI↑ through which it can be identified and individually referenced. Ontologies contain expert-curated domain knowledge↑ about specific kinds↑ of entities together with their properties and relations in the form of classes↑ defined through universal statements↑ (2,3). Ontologies in this sense do not include statements about particular entities (i.e., empirical data). (27)

OWL↑

The Web Ontology Language (OWL) is a set of knowledge representation languages for authoring semantic graphs↑. They are characterized by formal semantics and build upon RDF↑. OWL provides three increasingly expressive sub-languages, i.e. OWL Lite, OWL DL, and OWL Full, that support reasoning based on description logics↑ to varying degrees. (28,29)

OWL Manchester syntax↑

The Manchester syntax is a user-friendly compact syntax for OWL↑ 2 ontologies. It is easier to read for a human reader than other axiom-based syntaxes for OWL↑ 2. (30,31)

Parent class↑

Refers to the class↑ of which a given class is a direct subclass↑ of.

The class of all red things is the parent class of the class of all red balls.

Particular↑

Contrary to a universal↑, a particular (individual, token) is a real entity↑ that is always bound to a specific location in time and space.

Based on the primitive instantiation relation (32), which cannot be derived from any other relation, one can define a particular as anything that instantiates some universal↑ (33).

Your friend Bob, a particular cell, and a particular Pb atom are examples for particulars.

Perceptual non-conceptual content↑

Images, image stacks, 3D models, video and audio recordings of real entities↑ carry perceptual non-conceptual contents (i.e., image-based or audio-based information) that do not denote but rather demonstrate (see *aesthetic nonconceptual content* (10)). The meaning contained in a media item is not arbitrary but rests on a natural relation of resemblance to the part of reality that it reproduces (*natural meaning* (22,23)). Media are important for documenting scientific results because they carry complex spatial and temporal information that often cannot be represented using textual representational artifacts↑ (but see (34)). Moreover, contrary to words, a media item can – based on its natural relation of

resemblance – carry meaning independent of other representational artifacts[↑], and thus can possess validity by itself (35). Media can also increase the trustworthiness of data by functioning as objective proofs (36,37). Iconic representational artifacts thus take in a mediating role between real entities[↑] and their textual representational artifacts[↑]. They contain information that has to be transferred into a textual representation to become data (10).

Persistent identifier[↑]

A code that enables the unique naming of a digital resource (which, in turn, can refer to a physical object). The associated persistent identifier code is permanently recognizable and makes the resource permanently identifiable and findable.

Digital Object Identifiers (DOIs) are examples for persistent identifiers.

Proper name[↑]

A proper name is the textual representational artifact[↑] of a particular[↑]. (10,24)

'Large Hadron Collider' and 'Bob' are examples for proper names of a particular particle accelerator and a particular homo sapiens.

RDF[↑]

The Resource Description Framework (RDF) is a set of specifications of the World Wide Web Consortium (W3C) and is commonly used as a general method for modeling information using web resources. It provides a variety of syntax notations and data serialization formats and follows the general syntax of a triple statement[↑]. RDF plays an important role in knowledge management, ontologies, and the Semantic Web.(38)

Real entity[↑]

A real entity is an object, process, quality or state that exists in reality, independent of any human mind. Any given real entity is either a universal[↑] or a particular[↑]. (32,39)

Human being, cell, the chemical element Pb, Bob, a particular cell, and a particular Pb atom are examples for real entities.

Reference[↑]

Terms not only carry semantic conceptual content[↑] in the sense of a particular semantic value[↑], but they also have one or more referents. The reference of a proper name[↑] for instance is the particular[↑] that is the name bearer, whereas the reference of a kind term[↑] is all the particulars[↑] that instantiate the corresponding kind[↑]. Reference and semantic conceptual content[↑] are both important for the correct application of terminology. Only if you know what a term like 'cell' means and how a 'cell' can be identified, you can apply the term to correctly refer to a particular[↑] entity as a cell.

Referential lexical competence[↑]

One can distinguish referential from inferential lexical competence[↑]. The referential lexical competence of a person depends on the person's knowledge about the typical appearance of instances[↑] of a kind[↑], allowing them to recognize a portion of reality based on a given 'factual' description[↑]. It thus relates to empirical recognition criteria[↑] for a given term. Referential lexical competence can be differentiated into *naming referential competence*, which refers to a person's ability to select the right label and thus apply the correct name for a given particular object (object is given ➜ word must be found), and *application referential competence*, which refers to a person's ability to select the right particular object for a given label (word is given ➜ object must be identified) (40). Whereas inferential lexical competence[↑] relies on a semantic system, referential lexical competence relies on a perceptual and motor system. Although these two systems are distinct, they both interact (4).

Looking at the definition 'cell nucleus': "Organelle which has as its direct parts a nuclear membrane and nuclear matrix", a person possesses the relevant semantic inferential competence if they know the set of defining properties and thus the right part of the definition (i.e., definiens). Knowing which term is linked to this definition refers to the person's output referential competence and thus the left part of the definition (i.e., definiendum).

Representational artifact↑

We use two types of representational artifacts when communicating with others about real entities↑ and their cognitive representations↑. The purpose of representational artifacts is to induce cognitive representations↑ in the receiver that resemble those held by the sender—we want to share the same cognitive representation↑ when communicating about the same real entity↑. (10,11)

Semantic conceptual content↑

Textual representational artifacts↑ carry semantic conceptual content (i.e., text-based information) by using words. Words, in turn, are linguistic conventions and thus their meaning is determined by common agreement (10) (*non-natural meaning* (22)). Categories such as ‘true’ and ‘false’, as well as basic modes of reasoning such as deduction and induction, can be applied only to semantic conceptual content. Textual representational artifacts↑ are directly open to discourse. Unlike iconic representational artifacts↑, they carry content that can be communicated, recorded, documented, and organized. Therefore, data must take the form of textual representational artifacts↑.

Semantic graph↑

A semantic graph is a network of RDF↑/OWL↑-based triple↑ statements, in which a given URI↑ takes the *Object* position in one triple↑ and the *Subject* position in another triple↑, connecting the triples↑ to form a directed labeled graph. Because both ontologies and empirical data can be documented using RDF↑/OWL↑, one can distinguish class↑-based and instance↑-based semantic graphs, the former consisting of TBox expressions and the latter of ABox expressions. (41)

Semantic knowledge graph↑

In our understanding, semantic knowledge graphs consist of instance↑based semantic graphs↑ that reference class↑-based semantic graphs↑, as it is the case when representing empirical data in the form of assertional statements↑ represented in the form of a semantic graph↑ that references ontology class↑ terms. Due to conceptual and technical advantages of instance↑-based over class↑-based semantic graphs↑ (42), data and metadata should be represented as semantic knowledge graphs. Semantic knowledge graphs can be stored in a knowledge base↑.

Semantic value↑

The semantic value of a given term (either kind term↑ or proper name↑) or a statement (either universal↑ or assertional↑) refers to the meaning of the term and thus the semantic conceptual content↑ it carries. Semantic value can be specified by class↑ definitions.

Set↑

A grouping concept for defining a general group↑. A set is always defined extensionally by listing its elements (i.e. denotative definition). As a consequence, members of the same set do not necessarily have to share a common property except for being a member of this set. (6,7) We distinguish two types of set concepts, i.e. fuzzy sets↑ and sets based on Wittgenstein’s notion of family resemblance.

The set X comprising Bob, the football of my son, and the pencil on my desk.

Sister-class↑

Definition: Refers to all classes↑ that share the same direct parent class↑.

Examples: *The classes of all red balls and the class of all blue balls are sister classes as they share the same parent class, i.e. the class of all red things.*

Subclass↑

Refers to the class↑ of which a given class is a direct parent class↑ of.

The class of all red balls is the subclass of the class of all red things.

Taxonomy↑

A nested tree hierarchy of kind terms↑ and their corresponding classes↑ resulting from Aristotelian definitions↑.

The taxonomy of biological taxa, with species at the leaves and higher taxa at the nodes.

Terminological statement↑

Statements about linguistic items such as synonymy statements in a glossary or ontology. (2–4)
Simple eye is a synonym for ocellus and head capsule is a synonym for cranium.

Textual representational artifact↑

Cognitive representations↑ that have been translated into a publicly accessible and enduring form, for instance, in sentences that contain proper names↑, alphanumeric identifiers, and general terms, including numbers and equations. Their communication allows specific cognitive representations↑ to exist independently in various minds. Textual representational artifacts carry semantic conceptual content↑ (i.e., text-based information) by using words. Words, in turn, are linguistic conventions and thus their meaning is determined by common agreement (10) (*non-natural meaning* (22). We can distinguish between textual representational artifacts of particulars↑ in the form of proper names↑ and of universals↑ in the form of kind terms↑ (10,24). Besides that, four types of textual representational artifacts can be distinguished: universal statements↑, contingent statements↑, terminological statements↑, and assertional statements↑.

Triple/Tuple↑

A tuple is an ordered value collection of n values. A triple is a tuple consisting of three values. In RDF↑, a triple is a statement consisting of three elements, forming a *Subject* - *Predicate* - *Object* expression. This triple-syntax plays a central role in RDF↑. *Subject* represents the object to be described and takes the form of a URI↑, *Predicate* one of its properties to be described or a relation in which it stands to another object and also takes the form of a URI↑, and *Object* either the value (i.e., literal) of the property or another object (i.e., URI↑) to which it has the relation specified by the *Predicate*. Triple statements can be modeled as semantic graphs↑. (38)
Example: (*Subject* neuron) http://purl.obolibrary.org/obo/CL_0000540 - (*Predicat* subclass of) https://www.w3.org/TR/rdf-schema/#ch_subclassof - (*Object* eukaryotic cell) http://purl.obolibrary.org/obo/CL_0000255

Universal↑

A universal is a real entity↑ that is multiply located. It is a kind↑ or type of entity.
Based on the primitive instantiation relation (32), which cannot be derived from any other relation, one can define a universal as anything that is instantiated by some particular↑ (33).
Human being, cell, and the chemical element Pb are examples for universals.

Universal statement↑

Statements that are true for all instances↑ of a specific universal↑. In Description Logics↑, they are referred to as TBox expressions. Universal statements represent commonly accepted domain knowledge. (2–4)
Definitions of anatomical terms, as they are provided in glossaries or in anatomy ontologies in the form of class axioms, are examples for universal statements (e.g. ocellus).

URI↑

Definition: A Uniform Resource Identifier (URI) is a string of characters that follows a specific structure and unambiguously identifies a particular resource. The URI can also serve as a URL (web address), and can be resolved to an IP address (see the example URI below).
Example: http://purl.obolibrary.org/obo/CL_0000255 (for eukaryotic cell)

References

1. GBIF REST API summary [Internet]. Available from: https://www.gbif.org/developer/summary
2. Schulz S, Stenzhorn H, Boekers M, Smith B. Strengths and limitations of formal ontologies in the biomedical domain. Electron J Commun Inf Innov Health. 2009 März;3(1):31–45.
3. Schulz S, Jansen L. Formal ontologies in biomedical knowledge representation. IMIA Yearb Med Inform 2013. 2013 Jan;8(1):132–46.
4. Seppälä S, Ruttenberg A, Schreiber Y, Smith B. Definitions in Ontologies. Cah Lexicol. 2016;2016-

Janua(109):173–205.
5. Big data [Internet]. Available from: https://en.wikipedia.org/wiki/Big_data
6. Mahner M, Bunge M. Foundations of biophilosophy. Berlin, Heidelberg: Springer; 1997. 1–423 p.
7. Audi R. The Cambridge Dictionary of Philosophy, 2nd Edition. Cambridge: Cambridge University Press; 1999.
8. Winsor MP. Non-essentialist methods in pre-Darwinian taxonomy. Biol Philos. 2003;18(3):387–400.
9. Stamos DN. Pre-Darwinian Taxonomy and Essentialism – A Reply to Mary Winsor. Biol Philos. 2005;20(1):79–96.
10. Vogt L. Signs and terminology: science caught between language and perception. Bionomina. 2011;4:1–41.
11. Smith B, Kusnierczyk W, Schober D, Ceusters W. Towards a Reference Terminology for Ontology Research and Development in the Biomedical Domain. In: Bodenreider O, editor. Proceedings of KR-MED 2006, Studies in Health Technology and Informatics, Vol 124. IOS Press; 2006. p. 57–66.
12. Gray J. Jim Gray on eScience: A Transformed Scientific Method. In: Hey T, Tansley S, Tolle K, editors. The Fourth Paradigm: Data-Intensive Scientific Discoveries. Redmond, Washington: Microsoft Research; 2009. p. xvii–xxxi.
13. Lynch C. Jim Gray's Fourth Paradigm and the Construction of the Scientific Record. In: Hey T, Tansley S, Tolle K, editors. The Fourth Paradigm: Data-Intensive Scientific Discoveries. Redmond, Washington: Microsoft Research; 2009. p. 177–184.
14. Bohle S. What is E-science and How Should it be Managed? [Internet]. SciLogs, Scientific and Medical Libraries. 2013. Available from: http://www.scilogs.com/scientific_and_medical_libraries/what-is-e-science-and-how-should-it-be-managed/
15. Smith B, Rosse C. The role of foundational relations in the alignment of biomedical ontologies. Stud Health Technol Inform. 2004 Jan;107(Pt 1):444–8.
16. Vogt L. eScience and the need for data standards in the life sciences: in pursuit of objectivity rather than truth. Syst Biodivers. 2013 Sep;11(3):257–270.
17. Wilkinson MD, Dumontier M, Aalbersberg IJ, Appleton G, Axton M, Baak A, et al. The FAIR Guiding Principles for scientific data management and stewardship. Sci Data. 2016 Dezember;3(1):160018.
18. Jacobsen A, de Miranda Azevedo R, Juty N, Batista D, Coles S, Cornet R, et al. FAIR Principles: Interpretations and Implementation Considerations. Data Intell. 2019 Nov;(November):10–29.
19. Klaua D. Über einen Ansatz zur mehrwertigen Mengenlehre. Monatsblatt Dtsch Akad Wiss Zu Berl. 1965;7:859–876.
20. Zadeh LA. Fuzzy stets. Inf Control. 1965;8:338–353.
21. Zimmermann H-J. Fuzzy set theory - and its applications. Berlin: Springer; 2001. 514 p.
22. Grice HP. Meaning. Philos Rev. 1957;66(3):377–388.
23. Hanna R. Kant and Nonconceptual Content. Eur J Philos. 2005 Aug;13(2):247–290.
24. Russell B. On Denoting. Mind. 1905;14(56):479–93.
25. De Giacomo G, Lenzerini M. TBox and ABox Reasoning in Expressive Description Logics. In: Proceedings of the Fifth International Conference on Principles of Knowledge Representation and Reasoning (KR'96) [Internet]. Morgan Kaufmann; 1996. p. 316–327. Available from: http://www.aaai.org/Papers/Workshops/1996/WS-96-05/WS96-05-004.pdf
26. Smith B. Ontology. In: Floridi L, editor. Blackwell Guide to the Philosophy of Computing and Information. Oxford: Blackwell Publishing; 2003. p. 155–166.
27. Vogt L, Baum R, Bhatty P, Köhler C, Meid S, Quast B, et al. SOCCOMAS: a FAIR web content management system that uses knowledge graphs and that is based on semantic programming. Database. 2019 Jan;2019(baz067):1–22.
28. W3C: OWL 2 Web Ontology Language Manchester Syntax (Second Edition).
29. W3C OWL Web Ontology Language [Internet]. Available from: https://www.w3.org/TR/owl-features/
30. W3C: OWL 2 Web Ontology Language Manchester Syntax (Second Edition) [Internet]. Available from: https://www.w3.org/TR/owl2-manchester-syntax/
31. Horridge M, Drummond N, Goodwin J, Rector A, Wang HH. The Manchester OWL Syntax. In: Proceedings of the OWL Experiences and Directions Workshop (OWLED'06) at the ISWC'06. 2006.
32. Smith B. The Logic of Biological Classification and the Foundations of Biomedical Ontology. Spat Cogn Comput. 2004;25(2000):25–29.
33. Smith B. Beyond Concepts: Ontology as Reality Representation. In: Varzi A, Vieu L, editors. Proceedings of FOIS 2004 International Conference on Formal Ontology and Information Systems, Turin, 4-6 November 2004 [Internet]. 2004. p. 4–6. Available from: http://ontology.buffalo.edu/bfo/BeyondConcepts.pdf
34. Puget A, Mejino JLV, Detwiler LT, Franklin JD, Brinkley JF. Spatial-symbolic query engine in anatomy. Methods Inf Med. 2012 Dezember;51(6):463–78.
35. Vernant J-P. Mythos und Gesellschaft im alten Griechenland. Frankfurt am Main: Suhrkamp Verlag; 1987. 242 p.
36. Daston L, Galison P. The image of objectivity. Representations. 1992;(40):81–128.

37. Heintz B. Die Innenwelt der Mathematik: Zur Kultur und Praxis einer beweisenden Disziplin. Wien: Springer; 2000.
38. W3C: Resource Description Framework (RDF) [Internet]. Available from: https://www.w3.org/RDF/
39. Smith B. On substances, accidents and universals - In defence of a constituent ontology. Philos Pap. 1997;27:105–127.
40. Marconi D. On the Structure of Lexical Competence. Proc Aristot Soc. 1995 Jun 1;95(1):131–50.
41. Vogt L. Organizing phenotypic data—a semantic data model for anatomy. J Biomed Semant. 2019 Dezember;10(1):12.
42. Vogt L. Morphological Descriptions in times of eScience: Instance-Based versus Class-Based Semantic Representations of Anatomy.
